# Synthesis and antibacterial activity evaluation of N (7) position-modified balofloxacins

**DOI:** 10.3389/fchem.2022.963442

**Published:** 2022-08-19

**Authors:** Ge Hong, Weitian Li, Lina Mao, Jiawen Wang, Tianjun Liu

**Affiliations:** Tianjin Key Laboratory of Biomedical Materials, Institute of Biomedical Engineering, Chinese Academy of Medical Sciences and Peking Union Medical College, Tianjin, China

**Keywords:** fluoroquinolone, antimicrobial activities, cytotoxicity, hemolysis, balofloxacin

## Abstract

A series of small-molecule fluoroquinolones were synthesized, characterized by HRMS and NMR spectroscopy, and screened for their antibacterial activity against MRSA, *P. aeruginosa*, and *E. coli* as model G^+^/G^−^ pathogens. Compounds **2-e**, **3-e**, and **4-e** were more potent than the reference drug balofloxacin against MRSA and *P. aeruginosa* (MIC values of 0.0195 and 0.039 μg/ml for **2-e**, 0.039 and 0.078 μg/ml for each of **3-e** and **4-e**, respectively). Analysis of the time-dependent antibacterial effect of compound **2-e** toward MRSA showed that in the early logarithmic growth phase, bactericidal effects occurred, while in the late logarithmic growth phase, bacterial inhibition occurred because of concentration effects and possibly the development of drug resistance. Compound **2-e** exhibited low toxicity toward normal mammalian cell lines 3T3 and L-02 and tumor cell lines A549, H520, BEL-7402, and MCF-7. The compound was not hemolytic. Atomic force microscopy (AFM) revealed that compound **2-e** could effectively destroy the membrane and wall of MRSA cells, resulting in the outflow of the cellular contents. Docking studies indicated the good binding profile of these compounds toward DNA gyrase and topoisomerase IV. ADMET’s prediction showed that most of the synthesized compounds followed Lipinski’s “rule of five” and possessed good drug-like properties. Our data suggested that compound **2-e** exhibited potent anti-MRSA activity and is worthy of further investigation.

## Introduction

Fourth-generation quinolones, such as balofloxacin (Ansari et al., 2021; El-Azazy et al., 2021), moxifloxacin (Celebi and Onerci Celebi, 2021; Chen and Chang, 2021), and pazufloxacin (Khan et al., 2018), have broad-spectrum antibacterial activity and play an important role in anti-infective chemotherapy. With the emergence of multidrug-resistant bacterial strains (Alzahrani et al., 2022), the required working doses of these compounds are increasing, but patient tolerance of these drugs is relatively low. The discovery of novel, potent, antibacterial chemical entities based on quinolones would be an economical and relatively fast solution to these problems (Mentese et al., 2013). Many fluoroquinolone derivatives have been designed and synthesized (Donmez and Dogan, 2021; Jia and Zhao, 2021; Wallace et al., 2021), and their structure-activity relationships (SARs) have been revealed (Lai et al., 2005; Abdel-Aal et al., 2019).

The general essential structural characteristics of fluoroquinolone antibiotics are a carboxyl group at position C (3) and a carbonyl group at C (4), with five- or six-membered nitrogen heterocycles such as pyrrolidine, piperazine, and piperidine as fused ring structures. In the past few decades, structural modification reports have mainly focused on positions N (1), C (7), or C (8). Modification at C (7) is found to be most significant for the antibacterial efficacy, physical properties, bioavailability, and safety of fluoroquinolones/naphthyridinones (Borner et al., 1999; Monti et al., 2001; Araujo-Neto et al., 2021). The tricyclic quinolone molecular skeleton is essential (Huddar et al., 2020; Das et al., 2021; Gurung et al., 2022). Introduction of a phenyl (Baker et al., 2004), spliced pyrrole (Liu and Guo, 1992), triazole (Zhang et al., 2019), or imidazole group (De Francesco et al., 2021) to position N (1) could enhance the antibacterial activity. Changing the primary amine in fluoroquinolone to cyclic amines such as a piperazine, pyrrolidine, or piperidine, yield balofloxacin, moxifloxacin, and pazufloxacin respectively (Baiza-Duran et al., 2018; Miyashita et al., 2018; Baiza-Durán eta al., 2019). Notably, the bulkiness of the substituents at the C (7) position and the ensemble molecular mass are not obstacles to cell membrane penetration (Zhang et al., 2018). Therefore, installing different pharmacophores in this position would be helpful to find more potent compounds.

**FIGURE 1:**
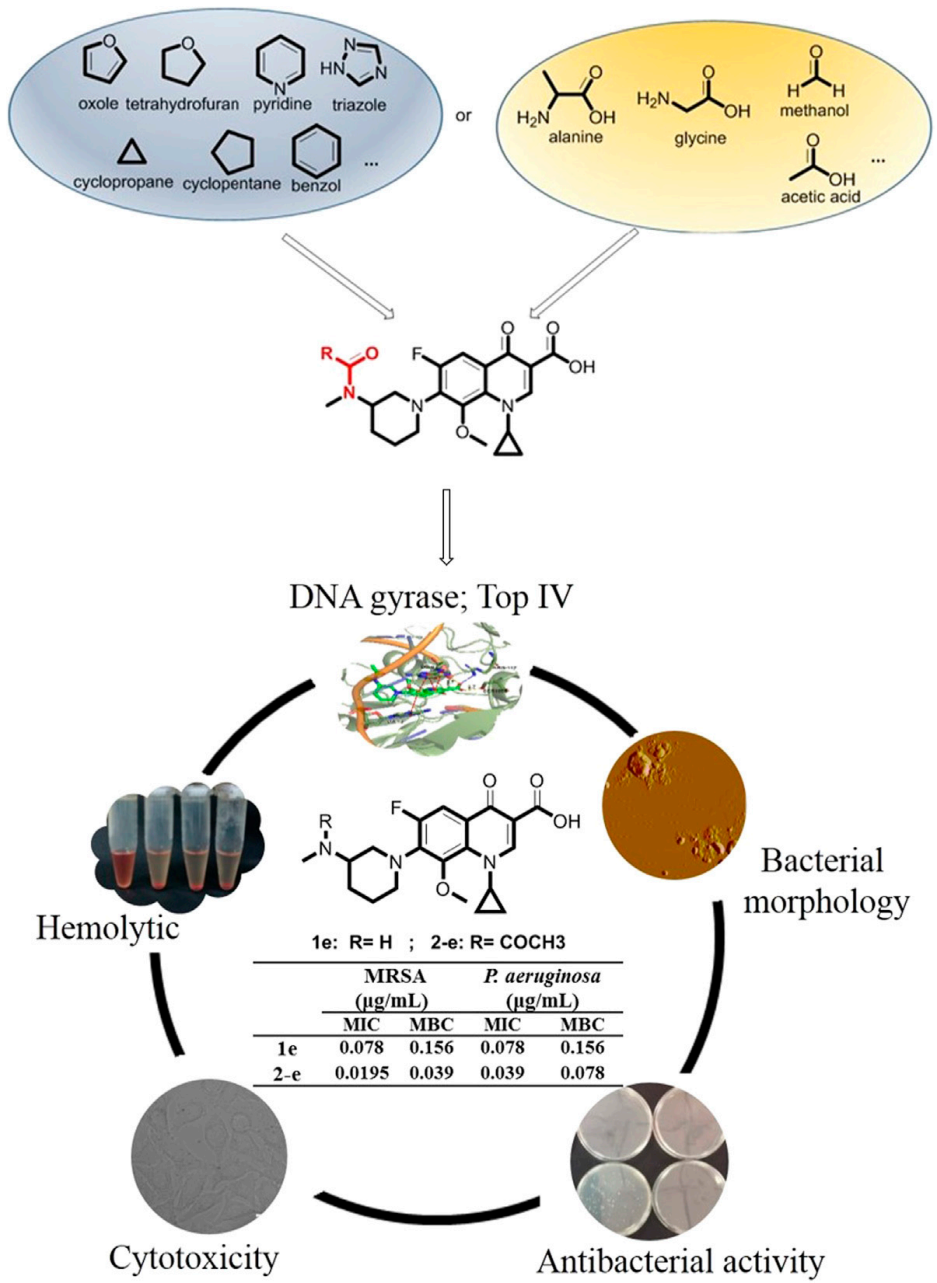
Strategies for synthesis and evaluation of the **N (7)** position-modified balofloxacins.

Balofloxacin represents a new generation of fluoroquinolone antibiotics. It can be used to treat urinary tract infections such as cystitis, urethritis, and other urinary system inflammation caused by *Staphylococcus*, *Streptococcus*, *Escherichia coli*, and other bacteria (Ansari et al., 2021; El-Azazy et al., 2021). Is it possible to improve its pharmacological properties by chemical modification? This study focused mainly on the small molecule groups on which fluoroquinolone compounds depend for their antibacterial activity. Modifiying groups were selected based upon the drug metabolism pathways in the human body, such as glycosylation, acetylation, and amino acidification (Ma et al., 2018). Sugar-substituted fluoroquinolones were reported previously and were not particularly potent (Hafez and El-Gazzar, 2015). Here, alkyl, aromatic, acetyl groups, partial amino acid moieties, and heterocyclic substituents such as triazole, which were used in antibacterial agents, were respectively introduced into balofloxacin. Acetyl was also introduced into moxifloxacin and pazufloxacin, while triazole was introduced into moxifloxacin. In total, 22 N (7) position-modified fluoroquinolones were obtained and their antibacterial activities were explored *in vitro* and *in silico*. Compound 2-e, which had the most potent antibacterial activity, was analyzed for time-dependent effects, its effect on bacterial morphology, and its biocompatibility *in vitro*. The strategies for synthesis and evaluation of the N (7) position-modified balofloxacins are outlined in Figure 1.

## Materials and methods

### Chemistry

All of the chemical reagents used were purchased from China. Reactions were monitored by thin-layer chromatography (TLC) on silica gel GF254 plates (Qingdao Haiyang Chemical Plant, Qingdao, China) and then visualized at 254 and 365 nm. Flash column chromatography was performed with silica gel (200–300 mesh). Melting points were determined on a digital melting point apparatus and were not corrected (Shengguang WRS-1B, Shanghai, China). High-resolution mass spectra (HR-MS) were recorded on Agilent 6520 QTOF LC/MS and Varian 7.0T FTMS (MALDI). ^1^H-NMR spectra and ^13^C-NMR were acquired on a Mercury Vx-300 (300 MHz) or Bruker AVANCE III (400 MHz) and referenced to tetramethylsilane (TMS). The purity of all the tested compounds was over 95% determined by HPLC; all of the biological tests were conducted in an ultra-clean workbench (SW-CJ-IFD, Suzhou). The synthesis experiment and structural elucidation of each compound can be found in detail in the Supplementary Material.

### Biological activity screening

#### MIC and MBC determinations *in vitro*

The three strains of MRSA, *P. aeruginosa,* and *E. coli* used in this experiment were from clinical isolates, which were provided by the People’s Liberation Army 304 Hospital.

To evaluate the antibacterial activities of newly synthesized N (7) position modified fluoroquinolones, the minimum inhibitory (MIC) and bactericidal (MBC) concentrations of these compounds toward bacteria were measured. The concentration of compounds to make the suspensions of bacteria from turbid to clarified by 24 h culture was regarded as MIC. The concentration makes the number of colonies no more than five, observed through solid medium culturing for 24 h was defined as MBC.

### Time-dependent antibacterial effects *in vitro*

In order to further verify the antibacterial effect of the representative compound **2-e**, time-dependent bactericidal activity of **2-e** against MRSA was determined. This study referred to the reported methods (Ling et al., 2015). MRSA was cultured overnight in LB liquid and the bacterial solution at 10^9^ CFU/ml tested on the machine was diluted at 1:10,000 with LB liquid medium and cultured at 37°C, 180 rpm. The bacteria were initially cultured for 2 h and 5 h, respectively, as the early and later time index, then interacted with 10 × MIC values of antibiotic (**1e**: 0.78 μg/ml; **2**-**e**: 0.20 μg/ml). Time-dependent bacterial colonies were evaluated at 4 or 8 h intervals as follows: 100 μL of the mixture at the current time point was taken and centrifuged at 10,000 rpm for 2 min, and the pellet formed was dispersed with PBS, which was diluted by the ten-fold dilution method. 100 μL of bacterial dilution was evenly spread on LB solid medium at 37°C for 24 h. The colonies counted, according to national standards, accounted for the drug’s time-dependent antibacterial results. The same experimental operation was repeated three times independently.

### Hemolytic activity

The animal experiment was approved by the Laboratory Animal Management Committee/Laboratory Animal Welfare Ethics Committee, Institute of Radiation Medicine, Chinese Academy of Medical Sciences (No. IRM-DWLL-2019153). Hemolysis anemia is one of the major adverse reactions of antibiotics. To assess the blood compatibility of N (7) position modified balofloxacin, the hemolytic activity of **2-e** was tested. Fresh rabbit blood, obtained from the ear vein of a male New Zealand white rabbit, was diluted at a ratio of 1:20 in volume with PBS, centrifuged and resuspended in PBS at 1,000 rpm for 5 min to form an experimental red cell suspension. 500 μL drug **2-e** solution and equal volumes of blood suspension were mixed in which the final drug concentration was 500, 250, 125, 62.5, 31.25, 15.62, 7.81, 3.91, 1.95, and 0.98 μg/ml, respectively, these mixtures were incubated at 37°C for 24 h. The positive control group was an equal volume of diluted blood cells mixed with an equal volume of 0.5% Triton X-100. The negative control group was mixed with an equal volume of PBS and diluted blood cells. After incubation, the mixture in a series of centrifuge tubes was centrifuged under the same conditions, and 200 μL of the supernatant was aspirated and transferred to a 96-well plate. The release of hemoglobin from the main contents was measured at A_405nm_ using a microplate reader (Thermo, Varioskan Flash 3001, United States). Each data were from the triplicate experiments. The percentage of hemolysis in the hemolysis test was calculated according to the following formula:

$$\%\text{Hemolysis}=\left({\text{A}}_{405\;\;\text{test sample}}-{\text{A}}_{405\text{ negative control}}\right)/\left({\text{A}}_{405\text{ positive control}}-{\text{A}}_{405\text{ negative control}}\right).$$

### Cell viability assays on 3T3, L-02, BEL-7402, H520, A549, and MCF-7

To investigate the biocompatibility of the synthesized balofloxacin derivative, the cytoxicity of **2-e** on human normal cells (3T3, L-02) and tumor cells (BEL-7402, H520, A549, and MCF-7) was studied. As an experimental example human liver cells (L-02) growing in the log phase, were plated in the 96-well plate at a density of 5000 cells/well. The cells were cultured at 37°C for 24 h under 5% CO_2_ medium. Then different concentrations of **1e** and **2-e** were added, the L-02 cells were cultured at 37°C for 48 h under a 5% CO_2_ atmosphere. Then the medium was aspirated, 100 μL of serum-free medium and 20 μL of MTS were added into each well, and L-02 was incubated at 37°C for 1 h under a 5% CO_2_ atmosphere. After that, the absorbance of the solutions at 490 nm was obtained using a microplate reader and calculated cell survival rate according to the following formula:

$$\text{Cell survive rate}\left(\%\right)=\left({\text{OD}}_{\text{test}}-{\text{OD}}_{\text{blank}}\right)/\left({\text{OD}}_{\text{control}}-{\text{OD}}_{\text{blank}}\right)\times 100\%.$$

#### Atomic force microscopic assay of sample preparation

To observe the structural changes in the bacterial surface after the administration of balofloxacin derivative **2-e**, atomic force microscope assay was performed. The bacterial suspension was diluted with LB liquid medium to 10^7^ CFU/ml. The bacterial solution and the drug solution were mixed in an equal volume so that the concentration of **1e** and **2-e** was 10 μM in the final solution, respectively. And after mixing, it was incubated at 37°C for 30 min, together with the control group in the same way. After that, the sample was centrifuged at 9,000 rpm for 5 min and the supernatant was discarded, while the precipitate was washed twice with physiological saline in the same volume and centrifuged at 9,000 rpm for 5 min. Finally, the bacterial sample, prepared by dispersion in the same volume of physiological saline solution, was dropped onto the surface of the mica plate (about 0.5 cm^2^), dried naturally at ambient temperature, and scanned by an atomic force microscope.

### Docking study

To rationalize the relationship between antibacterial activity and conformational preference for synthesized quinolones, docking calculations were performed with the Glide module of Mestro 11.5 (Schrödinger LLC, New York) (Friesner et al., 2006). The X-ray structures of DNA gyrase (PDB ID: 2XCT) and topoisomerase IV (PDB ID: 4KPF) with co-crystallized ligands were downloaded from the RCSB Protein Data Bank and preprocessed by fixing missing side-chain atoms, ionizing and tautomerizing het groups, optimizing hydrogen bonding networks, and removing water molecules with the Protein Preparation Wizard panel (Sastry et al., 2013). The binding site was confined to a 20 × 20 × 20 Å cuboid enclosing box with the centroid of coordinates (2XCT: *x =* 2.74, *y* = 44.49, *z* = 67.95; 4KPF: *x =* -40.34, *y* = 78.32, *z* = -10.99). The compounds were drawn using a 2D sketcher and prepared using the Ligprep module for low-energy 3D conformers. With the default parameters, flexible ligand docking was carried out using the extra precision (XP) mode (Bahekar et al., 2007). Docking accuracy was evaluated by extracting the bound conformation of the co-crystallized ligand and redocking into the same binding site. From the obtained results, docking score, glide energy, and glide ligand efficiency were finally chosen to rank the docking poses per ligand. After that, the binding free energy of each docking pose was calculated using the Prime MM-GBSA approach (Kekenes-Huskey et al., 2004), and the graphic models of compound **2-e** in complex with 2XCT/4KPF were generated by the 2D ligand interaction diagram and 3D Pose Viewer (Gupta et al., 2017) for detailed analysis of the receptor-ligand interaction.

### ADMET prediction *in silico*

Nowadays, many web-based tools are available to profile the physicochemical parameters and ADMET properties of drug candidates using *in silico* calculations. Here, new quinolone compounds were evaluated using two servers online, named Swiss ADME and admetSAR 2.0. Several physicochemical parameters such as molecular weight (MW), octanol-water partition coefficient (Log*P*), hydrogen bond donor (HBD), hydrogen bond acceptor (HBA), number of rotatable bonds (RBN), topological polar surface area (TPSA) and aqueous solubility (log*S*) were computed by Swiss ADME (Molecular Modeling Group, Swiss Institute of Bioinformatics, Lausanne, Switzerland, http://www.swissadme.ch/). Meanwhile, various ADMET properties, including human oral bioavailability (HOB), plasma protein binding (PPB), blood brain barrier (BBB), cytochrome P450 2D6 inhibition (CYP2D6), hepatotoxicity (HT) and carcinogenicity (Carcino) were predicted via admetSAR 2.0 (School of Pharmacy, East China University of Science and Technology, Shanghai, China, http://lmmd.ecust.edu.cn/admetsar2/).

## Results and discussion

### Chemistry

**SCHEME 1:**
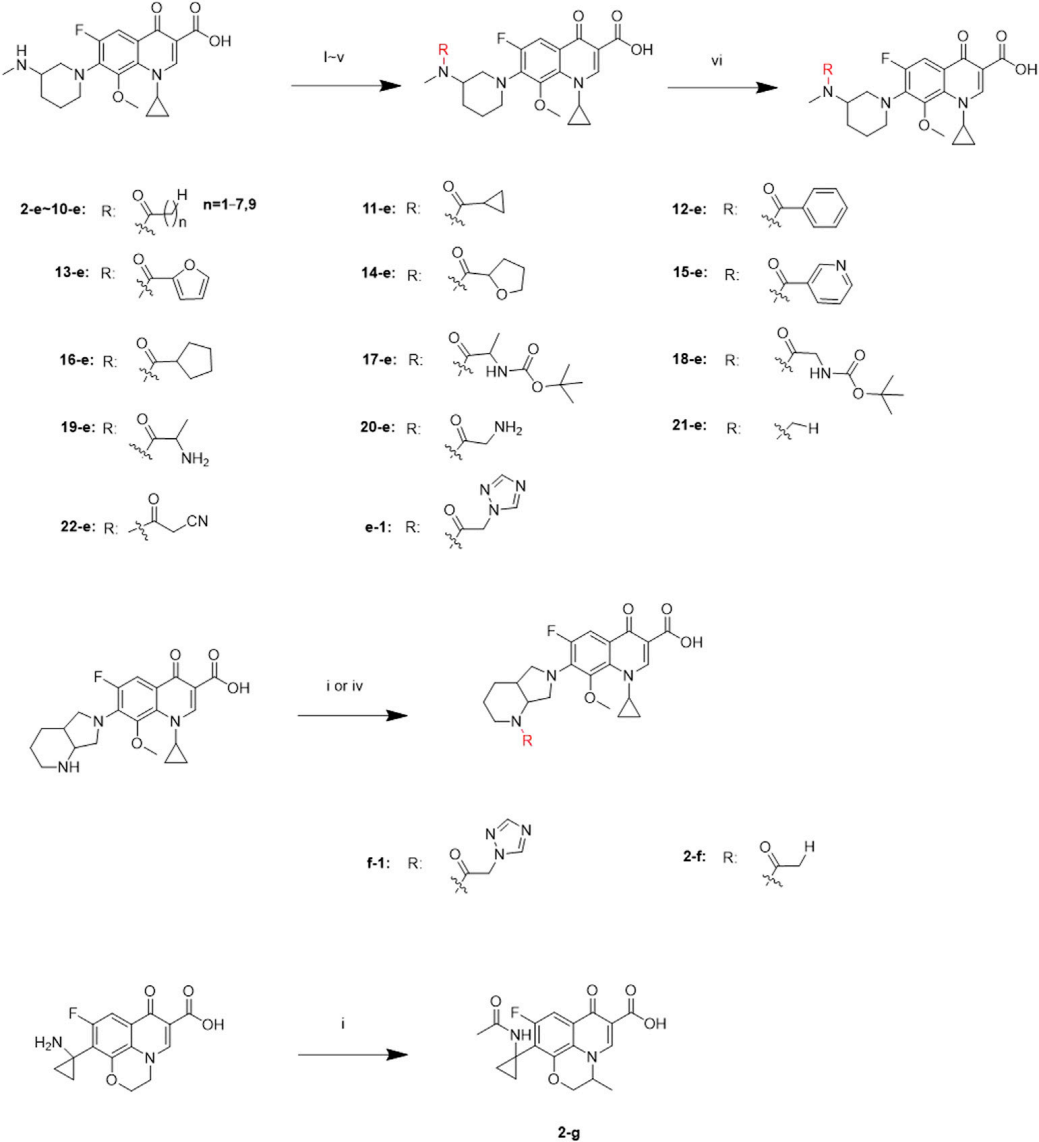
Synthesis of **2-e** ∼ **22-e**, **2-f**, **2-g**, **e-1,** and **f-1**. Reagents and conditions: (i) DMF, and TEA, r.t., 6–24 h; (ii) ethyl chloroformate, DMF, and TEA; r.t., 6–24 h; (iii) HCHO and HCOOH; 110°C, 30 h; (iv) the first step: chloroacetyl chloride, DMF, and TEA, 3–10 h; the second step: 1,2,4-triazole sodium, and CH_3_CN; 50°C, 48 h; (v) cyano-acetic acid, DMF, and TEA, 0°C, 24 h; (vi) TFA and CH_2_Cl_2_, r.t., 24 h.

**FIGURE 2:**
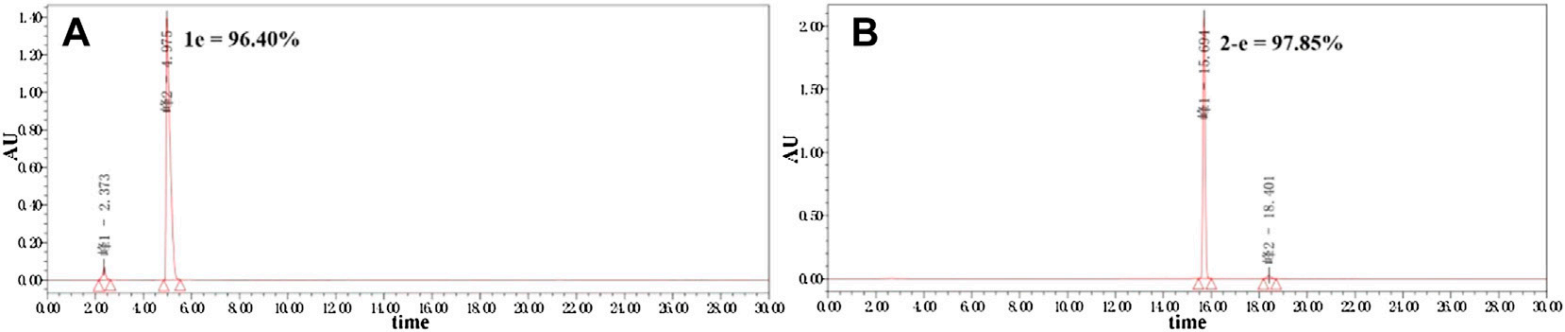
Purity of compounds **1e** and **2-e** analyzed by HPLC (Kromasil 100-5-C_18_ column 4.6 × 250 mm), gradient-eluted by A/B mixture solvent **(A):** acetonitrile; **(B):** 12.5 mmol/L aqueous phosphoric acid solution, 0–20 min, 25% A → 50% A, 20–30 min, and 50% A → 25% A; TEA adjusts the pH to about 3.0 at a flow rate of 1 ml/min, and absorbance recorded at 325 nm.

Twenty-two target compounds were synthesized (Scheme 1). Compounds **2-e** to **12-e** were obtained by the reaction of balofloxacin with corresponding substrates in DMF containing TEA. Moxifloxacin or pazufloxacin reacted with acetyl chloride to afford **2-f** and **2-g**. Compounds **13-e** to **18-e** were obtained from the reaction of balofloxacin with the corresponding mixed anhydrides, prepared from ethyl chloroformate in DMF with TEA. Compounds **19-e** and **20-e** were respectively synthesized by deprotection of **17-e** and **18-e** under TFA conditions in CH_2_Cl_2_. Methylation product **21-e** was obtained in a high yield by heating balofloxacin in a formaldehyde-containing formic acid solution. Cyano-acetyl chloride, obtained from the reaction of oxalyl chloride with cyanoacetic acid, reacted with balofloxacin to form compound **22-e**. Balofloxacin and moxifloxacin were chemically modified with chloroacetyl chloride, then the intermediates were reacted with 1,2,4-triazole sodium at 50°C for 48 h in CH_3_CN to form compounds **e-1** and **f-1**, respectively. The purity of all the synthesized compounds was ≥95%, detected by HPLC. The purity of compound **2-e** was 97.85% Figure 2.

All the newly synthesized fluoroquinolones were characterized by HRMS and NMR spectroscopy. Compound **2-e** was chosen to illustrate the process of structural elucidation: Compound **2-e**, 0.19 g was obtained, a pale-white powdery solid, yielding 86%. M.p. 201.6–202.3°C. ^1^H-NMR (300 MHz, CDCl_3_): 14.79 (s, 1H, -COOH); 8.78 (d, *J* = 3.7 Hz, 1H, -C_2_H); 7.81 (dd, *J* = 12.0, 9.0 Hz, 1H, -C_5_H); 4.78–4.68 (m, 1H, -NCH); 4.06–4.00 (m, 1H, 3′-Piperidine-H); 3.84–3.78 (m, 3H, -OCH_3_); 3.50–3.42 (m, 2H, 2′-Piperidine-H); 2.97–2.95 (d, *J* = 2.7 Hz, 3H,-NCH_3_); 2.90 (s, 1H, 2″-Piperidine-H); 2.87 (s, 1H, 2″-Piperidine-H); 2.11 (s, 3H,-COCH_3_); 1.93–1.82 (m, 4H, 3″-Piperidine-H, 4-Piperidine-H); 1.24–1.17 (m, 2H, -NCH_2_CH_2_); 1.04–0.96 (m, 2H,-NCH_2_CH_2_). ^13^C-NMR (100 MHz, CDCl_3_): 177.03 (C_4_O); 170.75 (-COCH_3_); 166.74 (-COOH); 156.58 (d, *J* = 251.7 Hz, -C_6_F); 149.84 (-C_2_); 145.77 (-C_8_O); 139.50 (C_7_); 133.81 (C_9_); 122.12 (C_10_); 108.01 (C_3_); 107.63 (C_5_); 62.30 (2′-Piperidine-C); 55.16 (3′-Piperidine-C); 52.24 (2″-Piperidine-C); 50.38 (-OCH); 40.59 (-NHCH); 31.35 (4-Piperidine-C), 28.69 (-NCH_3_), 26.67 (-COCH_3_), 22.10 (3″-Piperidine-C), 9.66 (NHCH_2_CH_2_); 9.37(NHCH_2_CH_2_). HR-MS (ESI): 432.1933 ([M + H]^+^, C_22_H_27_FN_3_O_5_^+^; calcd. 432.1935). The synthesis experiments and structural elucidation details of the other compounds are summarized in Supplementary Material.

### Determination of MIC and MBC *in vitro*

The antibiotic activities of various compounds are presented in Table 1. Compounds **2-e** to **10-e** were balofloxacin modified by different fatty acids. Among them, the antibacterial activity of compounds **2-e** to **4-e** toward MRSA was superior to that of compound **1e**. Compound **2-e** is the acetylation compound of **1e**. Notably, the MIC and MBC values toward MRSA changed from 0.078 to 0.156 μg/ml, respectively, for compound **1e,** to 0.0195 and 0.039 μg/ml, respectively, for compound **2-e**. For *P. aeruginosa*, the MIC and MBC values for compound **1e** were 0.078 and 0.156 μg/ml, respectively, compared with 0.039 and 0.078 μg/ml, respectively, for compound **2-e**.

**TABLE 1:** *In vitro* antimicrobial data as MIC (μg/ml) and MBC (μg/ml) for compounds.

Compound	MRSA		*P. aeruginosa*		*E. coli*	
	MIC	MBC	MIC	MBC	MIC	MBC
e-1	0.156	0.313	0.156	0.313	0.156	0.313
f-1	0.313	2.5	1.25	2.5	0.625	2.5
2-f	0.313	0.625	0.313	0.625	0.313	0.625
2-g	25	50	25	50	25	50
2-e	0.0195	0.039	0.039	0.078	2.5	5
3-e	0.039	0.078	0.078	0.078	5	10
4-e	0.039	0.078	0.078	0.078	5	20
5-e	0.078	0.156	0.078	0.156	10	20
6-e	0.156	0.625	0.313	0.625	20	20
7-e	0.313	0.625	0.625	1.25	>40	>40
8-e	1.25	5	1.25	5	>40	>40
10-e	5	20	5	20	>40	>40
11-e	0.039	0.078	0.078	0.156	10	20
12-e	0.078	0.313	0.156	0.313	20	20
13-e	0.078	0.156	0.078	0.156	10	20
14-e	0.078	0.156	0.156	0.313	20	20
15-e	0.078	0.156	0.078	0.156	10	20
16-e	0.313	0.625	0.313	0.625	20	40
19-e	1.56	3.125	0.39	1.56	12.5	25
20-e	25	50	0.78	6.25	25	50
21-e	0.078	0.313	0.313	0.313	0.625	0.625
22-e	0.39	0.78	0.78	1.56	0.78	1.56
1e (balofloxacin)	0.078	0.156	0.078	0.156	0.078	0.156
1f (moxifloxacin)	0.039	0.039	0.039	0.039	0.039	0.039
1g (pazufloxacin)	0.156	0.313	0.313	0.625	0.156	0.313

After acetylation of moxifloxacin or pazufloxacin to give compounds **2-f** and **2-g**, respectively, their activities were weaker than those of compounds **1f** and **1g**, respectively. In particular, compound **2-g** showed an obvious decrease in the antibacterial activity. The MIC and MBC values changed from 0.156 to 0.625 μg/ml for **1g** to 25–50 μg/ml for **2-g**. These data revealed that the activity of the acetylated fluoroquinolone compounds was tightly correlated to the mother structure, did not follow the same rule. In compounds **2-e** to **10-e**, modified with alkyl fatty acids, the bactericidal ability decreased gradually with the length of the alkyl chain. Compounds **11-e** to **16-e** were modified by saturated cyclic or aromatic moieties. Their MIC and MBC values changed in the order of **11-e** < **14-e** < **16-e** and **13-e** ≈ **15-e** < **12-e**. They showed the same trend as **2-e** to **10-e**; i.e., as the substituent group increased, the activity decreased. This was mainly because a large molecular skeleton size decreased the ability of the fluoroquinolone compounds to pass across the cell membrane and bind to their target site (Li et al., 2022).

The introduction of heteroatoms such as O or N could enhance the antimicrobial activity of the modified compounds. In addition, products with saturated cyclic modifications had higher antibacterial activity than those with unsaturated modifications. Amino acid modification decreases antibacterial potency. For example, for compounds **19-e** and **20-e**, the MIC and MBC values toward MRSA were 1.56 and 3.125 μg/ml, 25 and 50 μg/ml, respectively, compared with 0.078 and 0.156 μg/ml, respectively, for compound **1e**. This trend was the same in previous reports (Pokrovskaya et al., 2009; Cao et al., 2013; Fan et al., 2022). Methylated compound **21-e** (MIC and MBC 0.078 and 0.31 μg/ml, respectively), the cyano group-modified compound **22-e** (MIC and MBC 0.39 and 0.78 μg/ml, respectively), and the antibacterial group triazole-modified compound **e-1** (MIC and MBC 0.156 and 0.312 μg/ml, respectively) were less active toward MRSA than compound **1e** (MIC and MBC 0.078 and 0.156 μg/ml, respectively). In addition, triazole-modified compound **f-1** was poor compared with compound **1f** (MIC and MBC 0.039 and 0.039 μg/ml, respectively). Above all, acetylation product compound **2-e** exhibited the most potent antibacterial activity among the study compounds.

### Time-dependent antibacterial effects

**FIGURE 3:**
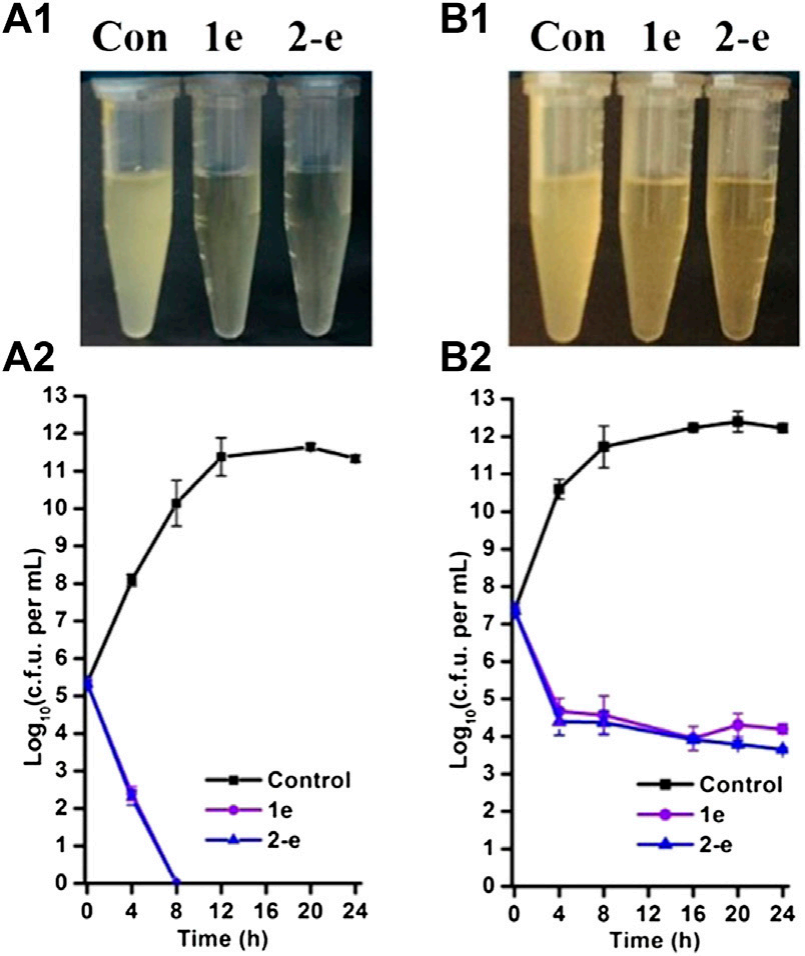
Time-dependent antibacterial effects by compounds **1e** and **2-e**. MRSA grown in early **(A)** and late **(B)** exponential phases challenged with antibiotics. Data were representative of three independent experiments ±s.d (A_1_ and B_1_ are the drug antibacterial results of 24 h in charts A_2_ and B_2_, respectively).

Compound **2-e** showed excellent antibacterial activity against MRSA (Table 1). In analysis of time-dependent antibacterial activity (Figure 3), at 10 × MIC, both **1e** and **2-e** showed good antibacterial activity in both the early and late stages of the exponential growth phase. This was consistent with the previously reported results for the commercially available antibiotics oxacillin and vancomycin (Ling et al., 2015). In the early exponential phase (i.e., at around log_10_ CFU = 5.5), the drug concentration per CFU was relatively high (at or near the MBC value), so the primary effect was bactericidal (Figure 3A_2_); log_10_ CFU decreased linearly in the first 8 h from 5.5 to 0. In the late exponential phase, when the number of bacterial CFU was high (around log_10_ CFU = 7.5), the drug concentration per CFU was relatively low (at or near the MIC value), so the dominating effect was bacterial inhibition. In these conditions, log_10_ CFU decreased from 7.5 to 4.3 in the first 4 h of drug action, and then only a small further decrease was observed up to 24 h (Figure 3B_2_). Therefore, the bactericidal action was mainly dependent upon the relative concentration of drug to the number of bacterial CFU. Long-term evolution would also be expected to produce drug-resistant.

### Hemolytic activity

**FIGURE 4:**
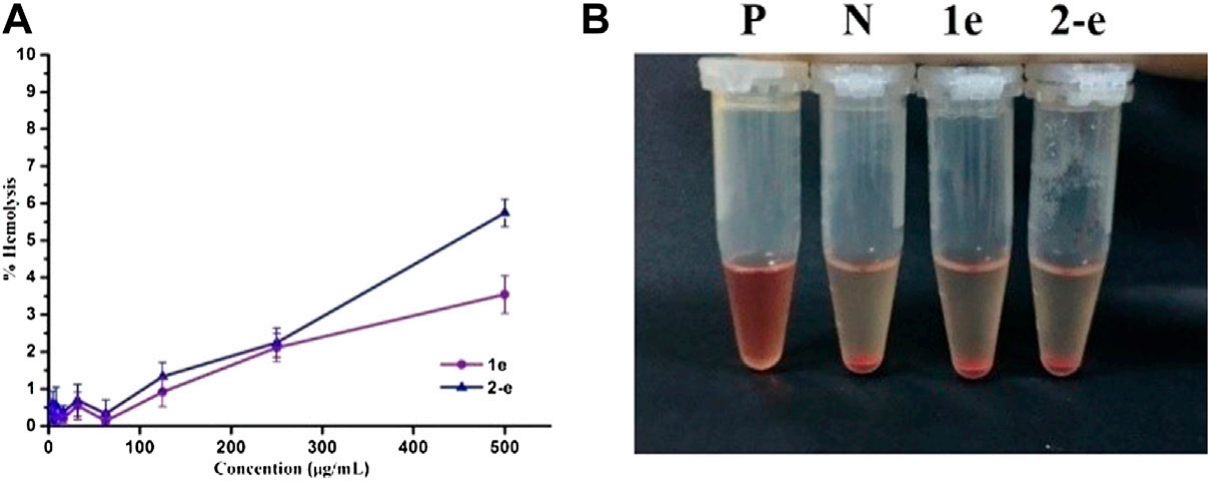
Hemolytic activity of **1e** and **2-e. (A)** Relationship between the hemolysis rate and concentration. **(B)** Hemolysis results at a concentration of 500 μg/ml (**P** and **N** positive and negative control, respectively).

Hemolytic assays of compounds **1e** and **2-e** were conducted to test their blood compatibility (Figure 4). The hemolysis rate of compound **2-e** was slightly higher than that of **1e**. The lipophilicity of **2-e** was slightly higher than that of **1e**, causing a relatively large amount to enter the red blood cell membrane. However, neither **1e** nor **2-e** had surfactant structures, so their hemolysis rates were very low (≤5%) even at high concentrations (e.g., > 10^5^ × MIC). This was different from the previously reported hemolytic fluoroquinolones temafloxacin and tosufloxacin (Rubinstein, 2001). As the working concentration of an antibacterial agent is much lower than 10^5^ × MIC in practice, almost no hemolysis effect can be expected in the application of compounds **1e** and **2-e**, they are safe antibacterial agents.

### Cytotoxicity analysis *in vitro* using 3T3, L-02, BEL-7402, H520, A549, and MCF-7 cell lines

**FIGURE 5:**
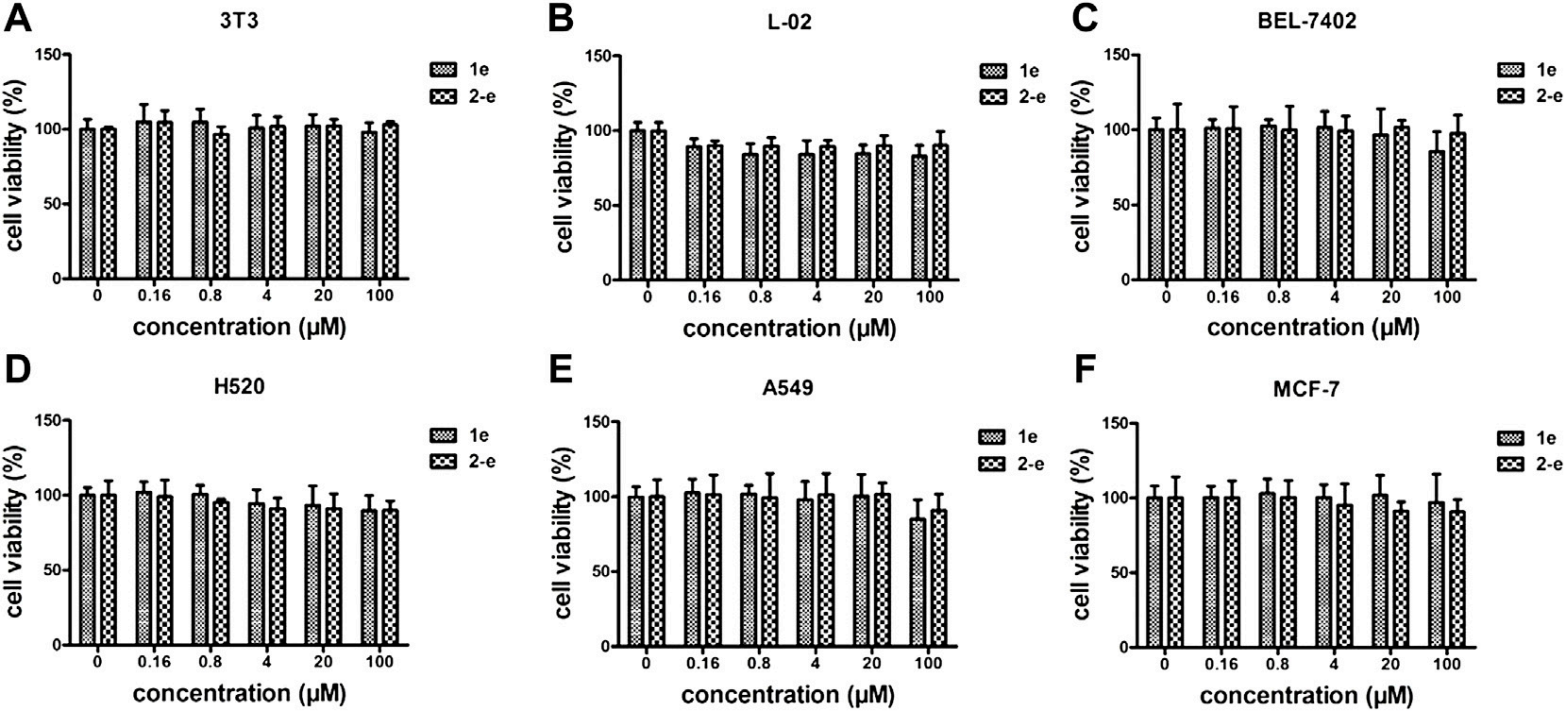
Cytotoxicity assay of **1e** and **2-e**. **(A)** 3T3; **(B)** L-02; **(C)** BEL-7402; **(D)** H520; **(E)** A549; **(F)** MCF-7.

As an antibacterial candidate compound (Chakraborty et al., 2021), the cytotoxicity of compound **2-e** toward normal mammalian cell lines 3T3 **(A)**, L-02 **(B),** and tumor cell lines BEL-7402**(C)**, H520**(D)**, A549**(E)**, and MCF-7**(F)** was investigated (Galarion et al., 2021) (Figure 5). Cell viability of compound **2-e** was ≥80% at 100 μmol/L in each case, implying that there was no significant toxicity toward these normal or tumor cells (Yang et al., 2022). The working concentration of **2-e** was expected to be much lower than 100 μmol/L (MIC and MBC <12 μmol/L), so it could be considered safe for eukaryotic cells. However, this finding was different from hepatotoxicity results using ADMET prediction (Table 3); the molecular structure of **2-e** contained a halogenated aromatic ring, which was assessed to be a toxic group by the computational chemistry platform.

**TABLE 3:** | ADMET properties of compounds.

Compound	Physicochemical properties								ADMET					
	MW^a^	Log*P* ^a^	HBD^a^	HBA^a^	RBN^a^	TPSA^a^	Log*S* ^a^	Rule of five	HOB^b^	PPB^b^	BBB^b^	CYP2D6^b^	HT^b^	Carcino^b^
e-1	498.51	0.97	1	8	8	122.79	−4.92	0	+	+	+	−	+	−
f-1	510.52	1.18	1	8	7	122.79	−4.95	1	+	+	+	−	+	−
2-f	443.47	1.63	1	6	5	92.08	−4.41	0	+	+	+	−	+	−
2-g	360.34	1.05	2	6	4	97.63	−3.39	0	+	+	+	−	+	−
2-e	431.46	1.42	1	6	6	92.08	−4.37	0	+	+	+	−	+	−
3-e	445.48	1.63	1	6	7	92.08	−4.86	0	+	+	+	−	+	−
4-e	459.51	1.84	1	6	8	92.08	−5.23	0	+	+	+	−	+	−
5-e	473.54	2.05	1	6	9	92.08	−5.79	0	+	+	+	−	+	−
6-e	487.56	2.25	1	6	10	92.08	−6.36	0	+	+	+	−	+	−
7-e	501.59	2.45	1	6	11	92.08	−6.92	2	+	+	+	−	+	−
8-e	515.62	2.65	1	6	12	92.08	−7.48	2	+	+	+	−	+	−
10-e	543.67	3.03	1	6	14	92.08	−8.60	2	+	+	+	−	+	−
11-e	457.49	1.84	1	6	7	92.08	−4.85	0	+	+	+	−	+	−
12-e	493.53	2.23	1	6	7	92.08	−6.09	0	+	+	+	−	+	−
13-e	483.49	1.08	1	7	7	105.22	−5.75	0	+	+	+	−	+	−
14-e	487.52	1.27	1	7	7	101.31	−4.99	0	+	+	+	−	+	−
15-e	494.51	1.25	1	7	7	104.97	−5.25	0	+	+	+	−	+	−
16-e	485.55	2.25	1	6	7	92.08	−5.97	0	+	+	+	−	+	−
19-e	460.50	0.85	2	7	7	118.10	−2.03	0	+	+	+	−	+	−
20-e	446.47	0.64	2	7	7	118.10	−1.61	0	+	+	+	−	+	−
21-e	403.45	1.66	1	6	5	75.01	−2.21	0	+	+	+	−	+	−
2; 2-e	456.47	0.78	1	7	7	115.87	−4.76	0	+	+	+	−	+	−
1e	389.42	1.44	2	6	5	83.80	−1.91	0	+	+	+	−	+	−
1f	401.43	1.66	2	6	4	83.80	−1.94	0	+	+	+	−	+	−
1 g	318.30	1.04	2	6	2	94.55	−0.70	0	+	+	+	−	+	−

a MW (molecular weight), Log*P* (lipophilicity), HBD (number of hydrogen bond donors), HBA (number of hydrogen bond acceptors), RBN (number of rotatable bonds), TPSA (topological polar surface area), and Log*S* (water solubility) values were calculated via SwissADME (Swiss Institute of Bioinformatics, http://www.swissadme.ch/). Rule of Five: number of violations of Lipinski’s rule of five. The rules are as follows: MW < 500, *c*log*P* < 5, HBD ≤5, HBA ≤10, and RBN ≤10.

b HOB (human oral bioavailability), PPB (plasma protein binding), BBB (blood–brain barrier), CYP2D6 (cytochrome P450 2D6 inhibition *in vitro*), HT (hepatotoxicity) (Mulliner et al., 2016), and Carcino (carcinogenicity) were predicted *via* admetSAR 2.0 (School of Pharmacy, East China University of Science and Technology, http://lmmd.ecust.edu.cn/admetsar2/). +, indicate positive result; −, indicate negative result.

### Cell wall damage monitored by atomic force microscopy (AFM)

**FIGURE 6:**
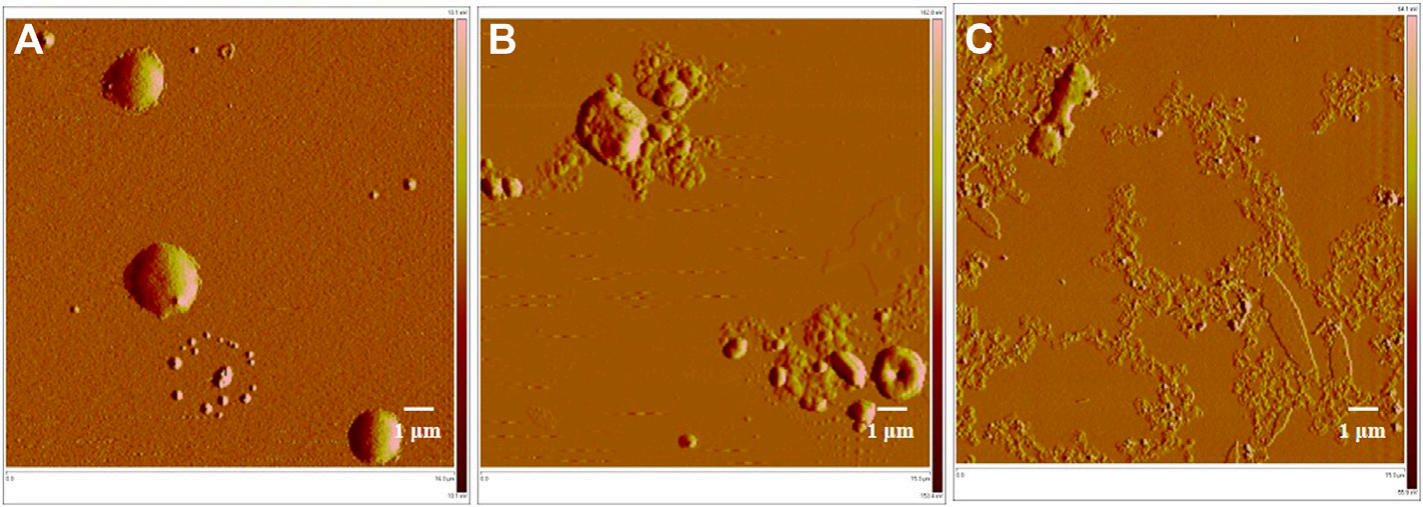
AFM image of MRSA bacterial strains; **(A)** control, 15 μm; **(B)**treated by 10 µM balofloxacin **1e**, 15 μm; and **(C)**treated by 10 µM acetylated quinolone **2-e**, 15 µm.

The ball-shaped structure of MRSA cells was observed in normal conditions (i.e., without any drug treatment) (Figure 6 a). After treatment with compound **1e**, this structure was ruptured and there was an outflow of cellular contents, but some of the collide-shape was kept (Figure 6 b). After treatment of MRSA cells with compound **2-e**, the extent of cellular breakage was much greater than that after treatment with **1e** (Figure 6 c); the cells showed complete discharge of their contents and substantial fragmentation. Although the molecular targets of fluoroquinolones were DNA gyrase and topoisomerase IV (Millanao et al., 2021; Piekarska et al., 2021; Shaheen et al., 2021), the AFM images revealed that compounds **1e** and **2-e** also caused MRSA cells to break into pieces. A possible interpretation of this phenomenon was that the pressure difference between the inside of the cells and the extracellular environment became large as the cells took up the drug. Finally, the high osmotic pressure led to disintegration of the cell (Hermawan and Putri, 2021). The AFM data confirmed that compound **2-e** was more potent than **1e**, as also shown by *in vitro* MIC and MBC values.

### Docking study

**FIGURE 7:**
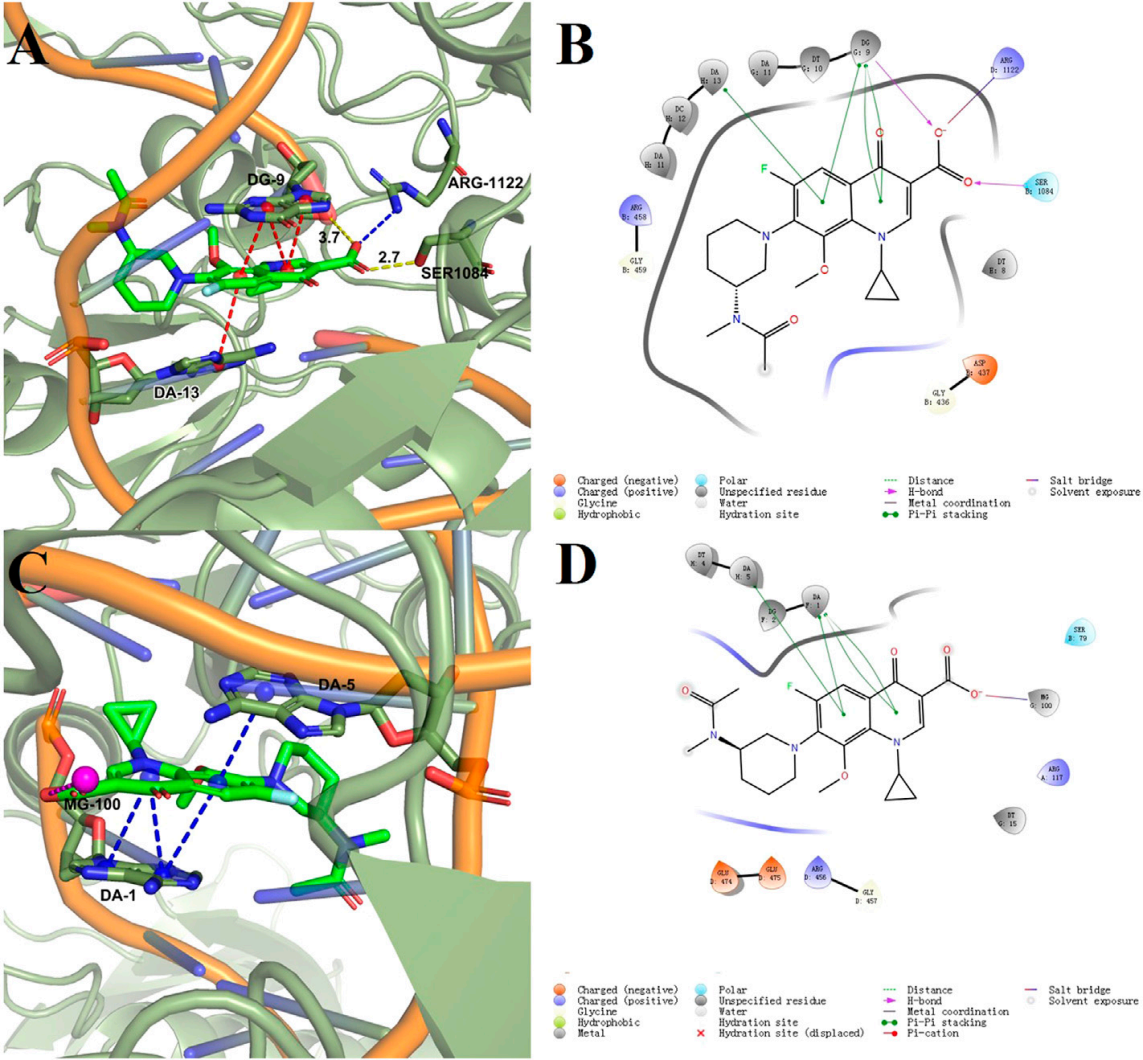
Docking models of compound **2-e** with DNA gyrase **(A,B)** and topoisomerase IV **(C,D)**.

Docking models provided a rational explanation for why compound **2-e** had strong inhibitory efficacy against target bacteria. Docking simulations were performed using the crystal structures of DNA gyrase and DNA topoisomerase IV as models (Ma et al., 2021). The orientation of **2-e** in 2D and 3D docking models was shown in Figure 7 π−π interactions between the fluoroquinolone core and residues of DNA gyrase (Figure 7B) or DNA topoisomerase IV (Figure 7D) made **2-e** lie planar in the pocket, while the 3D structure (Figure 7 A, C) revealed that **2-e** was embedded into DNA strands and interacted with residues (such as DA-13, DG-9, DA-1, and DA-5) via hydrogen bonding. Their interaction could inhibit the transcription and translation of DNA and cause bacterial death.

Docking score (DS), glide energy (GE), binding free energy (ΔG), and glide ligand efficiency (LE) (Zhang et al., 2020) for the compounds synthesized in this study were shown in Table 2. As reported previously (Stephen et al., 2022), the DS and GE were conventional indicators for evaluating molecular docking results between protein receptors and small molecule ligands. ΔG was the ensemble contribution of all the interaction surfaces; it increased with the molecular size. The LE reflected the average contribution of each ligand; it was a good indicator of the structure-activity relationship. Compared with the co-crystallized ligands, most of the synthesized fluoroquinolones showed higher DS and GE, while ΔG and LE were lower, which indicated higher affinity for the two target enzymes. This trend was consistent with the antibacterial activity data in Table 1. For a homogeneous series of compounds, such as **2-e** to **10-e**, the LE decreased with the length of the alkyl chain. The introduction of N or O in compounds **11-e** to **16-e** the LE decreased compared with compound **1e**. Modification of balofloxacin at **N (7)** with triazolyl, methyl, or other substituent groups affected the LE, namely the antibacterial activity. However, the antibacterial data were not always changed in the same way as the LE. Generally, different modification groups made the activity change differently.

**TABLE 2:** | Docking results of compounds with 2XCT and 4KPF

Compound	2XCT				4KPF			
	DS^a^	GE^a^	ΔG^a^	LE^a^	DS^a^	GE^a^	ΔG^a^	LE^a^
e-1	−4.92	−53.79	−58.60	−0.14	−8.87	−66.28	−53.81	−0.25
f-1	−6.13	−72.87	−60.03	−0.17	−7.48	−56.30	−48.41	−0.20
2-f	−6.25	−55.35	−47.26	−0.20	−7.96	−57.43	−48.02	−0.25
2-g	−6.92	−38.79	−49.26	−0.27	−9.19	−59.62	−51.60	−0.35
2-e	−5.67	−46.78	−56.93	−0.18	−8.03	−61.09	−43.82	−0.26
3-e	−5.49	−54.95	−53.79	−0.17	−8.07	−61.24	−49.21	−0.25
4-e	−5.56	−57.43	−57.42	−0.17	−7.73	−56.30	−53.40	−0.23
5-e	−5.37	−59.86	−63.82	−0.16	−7.56	−59.44	−58.05	−0.22
6-e	−5.54	−58.85	−61.37	−0.16	−7.66	−60.41	−59.31	−0.22
7-e	−5.25	−51.25	−58.93	−0.15	−8.21	−61.92	−55.58	−0.23
8-e	−5.22	−55.05	−60.98	−0.14	−7.31	−58.27	−47.14	−0.20
10-e	−4.64	−65.41	−47.65	−0.12	−8.27	−64.21	−56.57	−0.21
11-e	−5.35	−50.77	−57.72	−0.16	−7.83	−53.36	−53.37	−0.24
12-e	−5.23	−59.18	−50.27	−0.15	−7.75	−56.65	−52.19	−0.22
13-e	−5.68	−63.68	−55.86	−0.16	−7.85	−57.41	−53.71	−0.22
14-e	−4.78	−61.33	−52.64	−0.14	−8.09	−61.47	−54.22	−0.23
15-e	−5.79	−59.04	−59.69	−0.16	−8.01	−59.95	−59.25	−0.22
16-e	−5.33	−53.92	−72.06	−0.15	−7.78	−64.73	−50.86	−0.22
19-e	−7.93	−76.95	−52.47	−0.24	−9.92	−64.23	−53.79	−0.30
20-e	−9.21	−64.86	−62.47	−0.29	−10.35	−69.07	−45.89	−0.32
21-e	−7.08	−60.87	−45.57	−0.24	−10.93	−62.21	−53.17	−0.38
22-e	−5.89	−56.25	−54.89	−0.18	−8.69	−61.16	−47.19	−0.26
1e	−7.68	−63.58	−62.81	−0.27	−10.86	−59.12	−49.58	−0.39
1f	−8.50	−62.96	−56.06	−0.29	−9.79	−53.40	−44.64	−0.34
1g	−7.54	−43.11	−45.95	−0.33	−10.02	−49.82	−29.74	−0.44
IUV	—	—	—	—	−10.35	−60.62	−21.32	−0.38
CPF	−11.21	−66.58	−27.08	−0.47	—	—	—	—

a **DS** (docking score, kcal/mol), **GE** (glide energy, kcal/mol), **ΔG** (binding free energy, kcal/mol), and **LE** (glide ligand efficiency, kcal/mol).

### ADMET prediction

Prediction of ADMET (absorption, distribution, metabolism, excretion, and toxicity) properties (Amin et al., 2021; Daoud et al., 2021; Dwivedi et al., 2021) for all the target compounds was the same as that for compounds **1e**, **1f,** and **1g**, which indicated that modified compounds would be expected to have similar pharmaceutical properties to the original drugs. All the compounds except **f-1** and **7-e** to **10-e** met the requirements of Lipinski’s rule of five (their molecular weight ≤500 Da, lipophilicity, and the number of HBD ≤5, and the number of HBA and rotatable bonds ≤10). TPSA was a descriptor for evaluating the transportable properties of drugs in cells, and Log *S* was an indicator of the water solubility of compounds. As shown in Table 3, most of the synthesized fluoroquinolones had better membrane permeability but lower water solubility. This was consistent with the physicochemical properties of classical quinolone antibiotics (Sharma et al., 2016), and it seemed that salt formation was an effective way to increase the water solubility. In addition, these compounds possessed good predicted oral bioavailability, plasma protein binding rate, and blood–brain barrier permeability, lacked carcinogenicity, and inhibitory activity toward cytochrome P450 enzymes. Notably, all of them showed predicted hepatotoxicity, although in *in vitro* assays, compound **2-e** did not show toxicity toward L0-2 normal human liver cells even at 100 μmol/L (Figure 5B).

## Conclusion

A series of novel fluoroquinolones were synthesized and characterized, and their bacteriostatic and bactericidal activities were evaluated. Compounds **2-e**, **3-e**, and **4-e**, were more active than the reference drug balofloxacin (**1e**) against MRSA and *P. aeruginosa*. In particular, **2-e** showed potent antibacterial activity against MRSA with MIC values of 0.0195–0.039 μg/ml. Cell viability and hemolysis experiments indicated that compound **2-e** had good biocompatibility with mammalian cells. AFM revealed that compound **2-e** could destroy the cell walls and membranes of MRSA. Molecular docking studies indicated that compound **2-e** could interact with topoisomerase IV-DNA and DNA gyrase. Good ADMET data were obtained by *in silico* analysis. Overall, these results suggest that compound **2-e** was a candidate antimicrobial drug with medicinal potential that was worth studying further.

## Data Availability

The original contributions presented in the study are included in the article/Supplementary Material; further inquiries can be directed to the corresponding author.
