# Supplementary material for: Synthesis and antibacterial activity evaluation of N (7) position-modified balofloxacins
Source: Front Chem. 2022 Aug 19;10:963442. doi: 10.3389/fchem.2022.963442 (PMC9437215; doi:10.3389/fchem.2022.963442)
Supplement: Supplementary file 1 [file DataSheet1.docx]

Supplementary Material

**Synthetic experiments of compound 2-e to 12-e, 2-f and 2-g**

0.39 g (1.00 eq, 1.00 mmol) of parent compound **1e** was dissolved in 20 mL DMF, 150 μL (1.50 eq, 1.50 mmol) triethylamine and 155 μL (2.20 eq, 2.20 mmol) of acetyl chloride were slowly added dropwise, stirred in an ice-water bath at 0 ℃ for 30 minutes. Then, the solution was stirred for 5 h at room temperature. As the reaction was completed, 40 - 60 mL of deionized water was added, then extracted with dichloromethane three times, combined the extracts and evaporation, finally doping diethyl ether afforded 0.35 g target compound **2-e** as pale yellow powdery solid, the yield was 81%.

Compound **3-e** to **12-e, 2-f** and **2-g** were synthesized in the same way as compound **2-e**, in the yields of 70 - 90%.

*1-Cyclopropyl-6-fluoro-8-methoxy-7-(3-(N-methylacetamido)piperidin-1-yl)-4-oxo-1,4-dihydroquinoline-3-carboxylic acid* **(2-e)**

Compound **2-e**, 0.19 g was obtained, pale yellow powdery solid, yield 86%. M.p. 201.6-202.3 ℃. ^1^H-NMR (300 MHz, CDCl_3_): 14.79 (s, 1H); 8.78 (d, *J* = 3.7 Hz, 1H); 7.81 (dd, *J* = 12.0, 9.0 Hz, 1H); 4.78 - 4.68 (m, 1H); 4.06 - 4.00 (m, 1H); 3.84 - 3.78 (m, 3H); 3.50 - 3.42 (m, 2H); 2.97 - 2.95 (d, *J* = 2.7 Hz, 3H); 2.90 (s, 1H); 2.87 (s, 1H); 2.11 (s, 3H); 1.93 - 1.82 (m, 4H); 1.24 - 1.17 (m, 2H); 1.04 - 0.96 (m, 2H). ^13^C-NMR (100 MHz, CDCl_3_): 177.03; 170.75; 166.74; 156.58 (d, *J* = 251.7 Hz); 149.84; 145.77; 139.50; 133.81; 122.12; 108.01; 107.63; 62.30; 55.16; 52.24; 50.38; 40.59; 31.35; 28.69; 26.67; 22.10; 9.66; 9.37. HR-MS (ESI): 432.1933 ([M+H]^+^, C_22_H_27_FN_3_O_5_^+^; calcd. 432.1935).

*7-(1-Acetyloctahydro-6H-pyrrolo[3,4-b]pyridin-6-yl)-1-cyclopropyl-6-fluoro-8-methoxy-4-oxo-1,4-dihydroquinoline-3-carboxylic acid* **(2-f)**

Compound **2-f**, 0.33 g was obtained, light yellow powdery solid, yield 75%. M.p. 150.3-151.5 ℃. ^1^H-NMR (300 MHz, CDCl_3_): 15.01 (s, 1H); 8.77 (d, *J* = 3.4 Hz, 1H); 7.78 (d, *J* = 13.9, 9.0 Hz, 1H); 5.31 - 5.23 (m, 1H); 4.66 - 4.51 (m, 1H); 4.13 - 4.08 (m, 1H); 3.83 - 3.80 (m, 1H); 3.61 - 3.59 (m, 3H); 3.52 - 3.48 (m, 1H); 3.41 - 3.38 (m, 1H); 3.29 - 3.23 (m, 1H); 2.34 - 2.27 (m, 1H); 2.22 - 2.18 (m, 3H); 1.96 (br, 1H); 1.89 - 1.86 (m, 2H); 1.63 - 1.56 (m, 2H); 1.35 - 1.25 (m, 2H); 1.15 - 1.09 (m, 2H). HR-MS (ESI): 444.1934 ([M+H]^+^, C_23_H_27_FN_3_O_5_^+^; calcd. 444.1935).

*10-(1-Acetamidocyclopropyl)-9-fluoro-3-methyl-7-oxo-2,3-dihydro-7H-[1,4]oxazino[2,3,4-ij]quinoline-6-carboxylic acid* **(2-g)**

Compound **2-g**, 0.30 g was obtained, white powdery solid, yield 83%. M.p. 273.1-274.1 ℃, ^1^H-NMR (300 MHz, CDCl_3_): 14.73 (s, 1H); 8.69 (d, *J* = 9.4 Hz, 1H); 7.73 (d, *J* = 10.1 Hz, 1H); 6.29 (s, 1H); 4.57 (m, 2H); 4.50 - 4.46 (m, 1H); 1.88 (s, 3H); 1.67 (d, *J* = 6.5 Hz, 3H); 1.36 - 1.33 (m, 2H); 1.30 -1.27 (m, 2H). HR-MS (ESI): 361.1193 ([M+H]^+^, C_18_H_18_FN_2_O_5_^+^; calcd. 361.1200).

*1-Cyclopropyl-6-fluoro-8-methoxy-7-(3-(N-methylpropionamido)piperidin-1-yl)-4-oxo-1,4-dihydroquinoline-3-carboxylic acid* **(3-e)**

Compound **3-e**, 0.20 g was obtained, light yellow oil, yield 87%. ^1^H-NMR (300 MHz, CDCl_3_): 14.76 (s, 1H); 8.79 (d, *J* = 3.7 Hz, 1H); 7.84 (t, *J* = 11.6 Hz, 1H); 4.81 - 4.70 (m, 1H); 4.06 - 4.02 (m, 1H); 3.86 - 3.78 (m, 3H); 3.50 - 3.41 (m, 2H); 3.27 - 3.20 (m, 1H); 3.09 - 3.07 (m, 1H); 2.95 -2.91 (m, 3H); 2.51 - 2.43 (m, 1H); 2.39 - 2.32 (m, 1H); 1.90 -1.87 (m, 3H); 1.73 - 1.67 (m, 1H); 1.30 - 1.22 (br, 2H); 1.20 - 1.13 (m, 3H); 1.05 - 0.96(m, 2H). ^13^C-NMR (100 MHz, CDCl_3_): 177.08; 173.89; 166.79; 156.74 (d, *J* = 250.1 Hz); 149.86; 146.11; 139.50; 133.75; 122.24; 108.26; 107.86; 62.24; 53.16; 51.40; 50.65; 40.63; 30.93; 30.42; 28.71; 26.64; 9.66; 9.35; 9.22. HR-MS (ESI): 446.2090 ([M+H]^+^, C_23_H_29_FN_3_O_5_^+^; calcd. 446.2091).

*1-Cyclopropyl-6-fluoro-8-methoxy-7-(3-(N-methylbutyramido)piperidin-1-yl)-4-oxo-1,4-dihydroquinoline-3-carboxylic acid* **(4-e)**

Compound **4-e**, 0.19 g was obtained, light yellow oil, yield 83%. ^1^H-NMR (300 MHz, CDCl_3_): 14.77 (s, 1H); 8.77 (d, *J* = 3.6 Hz, 1H); 7.81 (t, *J* = 11.8 Hz, 1H); 4.78 - 4.71 (m, 1H); 4.06 - 4.00 (m, 1H); 3.85 - 3.78 (m, 3H); 3.49 - 3.40 (m, 2H); 3.30 - 3.02 (m, 2H); 2.95 - 2.89 (m, 3H); 2.45 - 2.39 (m, 1H); 2.34 - 2.27 (m, 2H); 1.94 - 1.86 (m, 2H); 1.74 - 1.58 (m, 3H); 1.24 - 1.16 (m, 2H); 1.01 - 0.98 (m, 2H); 0.96 - 0.93 (m, 3H). ^13^C-NMR (100 MHz, CDCl_3_): 177.05; 173.19; 166.75; 156.67 (d, *J* = 251.8 Hz); 149.96; 146.03; 139.59; 133.79; 122.14; 108.18; 107.79; 62.27; 54.22; 52.25; 50.55; 40.61; 36.08; 35.58; 30.60; 26.78; 18.38; 13.90; 9.66; 9.35. HR-MS (ESI): 460.2246 ([M+H]^+^, C_24_H_31_FN_3_O_5_^+^; calcd. 460.2248).

*1-Cyclopropyl-6-fluoro-8-methoxy-7-(3-(N-methylpentanamido)piperidin-1-yl)-4-oxo-1,4-dihydroquinoline-3-carboxylic acid* **(5-e)**

Compound **5-e**, 0.21 g was obtained, light yellow oil, yield 86%. ^1^H-NMR (300 MHz, CDCl_3_): 14.74 (s, 1H); 8.78 (s, 1H); 7.83 (t, *J* = 11.5 Hz, 1H); 4.79 - 4.71 (m, 1H); 4.04 - 4.02 (m, 1H); 3.86 - 3.72 (m, 3H); 3.50 - 3.41 (m, 2H); 3.30 - 3.07 (m, 2H); 2.96 - 2.88 (m, 3H); 2.47 - 2.30 (m, 2H); 2.04 - 1.84 (m, 3H); 1.65 - 1.60 (m, 3H); 1.43 - 1.33 (m, 2H); 1.26 - 1.19 (m, 2H); 1.06 - 0.99 (m, 2H); 0.96 - 0.91 (m, 3H). ^13^C-NMR (100 MHz, CDCl_3_): 177.03; 170.75; 166.74; 156.68 (d, J = 250.8 Hz); 149.84; 146.02; 139.51; 133.81; 122.19; 108.40; 107.80; 62.47; 54.48; 52.25; 50.72; 40.66; 33.88; 33.35; 30.77; 28.75; 27.42; 27.42; 13.82; 9.66; 9.36. HR-MS (ESI): 474.2402 ([M+H]^+^, C_25_H_33_FN_3_O_5_^+^; calcd. 474.2404).

*1-Cyclopropyl-6-fluoro-8-methoxy-7-(3-(N-methylhexanamido)piperidin-1-yl)-4-oxo-1,4-dihydroquinoline-3-carboxylic acid* **(6-e)**

Compound **6-e**, 0.20 g was obtained, light yellow oil, yield 80%. ^1^H-NMR (300 MHz, CDCl_3_): 14.75 (s, 1H); 8.71 (s, 1H); 7.71 (t, *J* = 10.7 Hz, 1H); 4.74 - 4.66 (m, 1H); 4.02 - 4.01 (m, 1H); 3.81 - 3.76 (m, 3H); 3.46 - 3.36 (m, 2H); 3.27 - 2.99 (m, 2H); 2.92 - 2.85 (m, 3H); 2.31 - 2.22 (m, 2H); 1.90 - 1.83 (m, 3H); 1.67 - 1.54 (m, 3H); 1.29 - 1.25 (m, 4H); 1.21 - 1.16 (m, 2H); 1.04 - 0.93 (m, 2H); 0.86 - 0.82 (m, 3H). ^13^C-NMR (100 MHz, CDCl_3_): 177.05; 173.49; 166.77; 156.62 (d, *J* = 251.9Hz); 149.93; 145.85; 139.59; 133.90; 121.96; 108.11; 107.62; 62.52; 54.46; 52.21; 50.71; 40.70; 34.07; 31.66; 31.29; 30.66; 28.74; 26.79; 23.53; 13.90; 9.83; 9.37. HR-MS (ESI): 488.2558 ([M+H]^+^, C_26_H_35_FN_3_O_5_^+^; calcd. 488.2561).

*1-Cyclopropyl-6-fluoro-8-methoxy-7-(3-(N-methylheptanamido)piperidin-1-yl)-4-oxo-1,4-dihydroquinoline-3-carboxylic acid* **(7-e)**

Compound **7-e**, 0.23 g was obtained, light yellow oil, yield 89%. ^1^H-NMR (300 MHz, CDCl_3_): 14.81 (s, 1H); 8.78 (d, *J* = 3.4 Hz, 1H); 7.81 (t, *J* = 11.9 Hz, 1H); 4.78 - 4.70 (m, 1H); 4.06 - 4.00 (m, 1H); 3.86 - 3.78 (m, 3H); 3.49 - 3.40 (m, 2H); 3.30 - 3.02 (m, 2H); 2.95 - 2.89 (m, 3H); 2.35 - 2.29 (m, 3H); 1.89 - 1.86 (m, 2H); 1.63 - 1.59 (m, 4H); 1.37 - 1.35 (m, 2H); 1.28 (br, 4H); 0.89 - 0.87 (m, 3H); 0.85 (br, 3H). ^13^C-NMR (100 MHz, CDCl_3_): 178.65; 173.62; 166.93; 156.69 (d, *J* = 252.1 Hz); 149.98; 146.06; 139.54; 133.86; 122.09; 108.30; 107.78; 62.48; 54.50; 52.24; 50.62; 40.62; 34.13; 33.52; 31.55; 30.64; 29.19; 26.55; 26.19; 22.50; 14.03; 9.83; 9.36. HR-MS (ESI): 502.2716 ([M+H]^+^, C_27_H_37_FN_3_O_5_^+^; calcd. 502.2717).

*1-Cyclopropyl-6-fluoro-8-methoxy-7-(3-(N-methyloctanamido)piperidin-1-yl)-4-oxo-1,4-dihydroquinoline-3-carboxylic acid* **(8-e)**

Compound **8-e**, 0.23 g was obtained, light yellow solid, yield 83%. M.p. 68.2-70.0 ℃. ^1^H-NMR (300 MHz, CDCl_3_): 14.81 (s, 1H); 8.77 (d, *J* = 3.1 Hz, 1H); 7.80 (t, *J* = 11.8 Hz, 1H); 4.78 - 4.70 (m, 1H); 4.05 - 4.02 (m, 1H); 3.85 - 3.78 (m, 3H); 3.49 - 3.40 (m, 2H); 3.30 - 3.02 (m, 2H); 2.95 - 2.89 (m, 3H); 2.35 - 2.29 (m, 3H); 1.94 - 1.86 (m, 2H); 1.68 - 1.56 (m, 4H); 1.29 (br, 2H); 1.27 - 1.26 (m, 6H); 0.88 - 0.87 (m, 3H); 0.86 - 0.84 (m, 3H). ^13^C-NMR (100 MHz, CDCl_3_): 177.84; 173.55; 166.83; 156.69 (d, *J* = 252.1 Hz); 149.98; 146.06; 139.60; 133.79; 122.13; 108.09; 107.51; 62.48; 53.82; 51.09; 50.63; 40.63; 34.12; 31.69; 30.79; 29.48; 27.81; 25.79; 24.94; 22.62; 22.60; 14.06; 9.75; 9.37. HR-MS (MALDI): 516.2870 ([M+H]^+^, C_28_H_39_FN_3_O_5_^+^; calcd. 516.2874).

*1-Cyclopropyl-6-fluoro-8-methoxy-7-(3-(N-methyldecanamido)piperidin-1-yl)-4-oxo-1,4-dihydroquinoline-3-carboxylic acid* **(10-e)**

Compound **10-e**, 0.25 g was obtained, white solid, yield 89%. M.p. 65.2-68.1 ℃. ^1^H-NMR (300 MHz, CDCl_3_): 14.84 (s, 1H); 8.81 (s, 1H); 7.86 (t, *J* = 12.4 Hz, 1H); 4.80 - 4.72 (m, 1H); 4.05 - 4.03 (m, 1H); 3.87 - 3.79 (m, 3H); 3.50 - 3.42 (m, 2H); 3.31 - 3.08 (m, 2H); 2.97 - 2.91 (m, 3H); 2.37 - 2.35 (m, 3H); 1.90 - 1.88 (m, 2H); 1.66 -1.59 (m, 4H); 1.31 (br, 2H); 1.27 (br, 10H); 0.91 - 0.90 (m, 3H); 0.89 - 0.86 (m, 3H). ^13^C-NMR (100 MHz, CDCl_3_): 178.43; 173.77; 167.08; 156.86 (d, *J* = 251.1 Hz); 150.07; 146.26; 139.69; 133.95; 122.34; 108.34; 107.79; 62.59; 54.64; 53.46; 51.12; 40.72; 34.26; 32.00; 30.78; 29.58; 29.39; 29.22; 27.77; 25.78; 25.24; 24.87; 22.81; 14.24; 9.88; 9.51. HR-MS (ESI): 544.3185 ([M+H]^+^, C_30_H_43_FN_3_O_5_^+^; calcd. 544.3187).

*1-Cyclopropyl-6-fluoro-8-methoxy-7-(3-(N-methylcyclopropanecarboxamido)piperidin-1-yl)-4-oxo-1,4-dihydroquinoline-3-carboxylic acid* **(11-e)**

Compound **11-e**, 0.20 g was obtained, white solid, yield 85%. M.p. 104.6-106.5 ℃. ^1^H-NMR (300 MHz, CDCl_3_): 14.76 (s, 1H); 8.78 (s, 1H); 7.82 (d, *J* = 12.1 Hz, 1H); 4.76 - 4.69 (m, 1H); 4.07 - 4.00 (m, 1H); 3.84 - 3.79 (m, 3H); 3.51 - 3.41 (m, 2H); 3.31 - 3.23 (m, 1H); 3.13 (br, 3H); 2.96 - 2.92 (m, 1H); 2.05 -1.90 (m, 4H); 1.76 (br, 1H); 1.25 - 1.21 (m, 2H); 1.00 (br, 4H); 0.85 - 0.78 (m, 2H). ^13^C-NMR (100 MHz, CDCl_3_): 177.05; 173.70; 166.77; 156.72 (d, *J* = 252.4 Hz); 149.86; 146.06; 139.56; 133.77; 122.20; 108.10; 107.65; 62.49; 54.36; 53.10; 51.22; 40.63; 30.79; 28.29; 25.78; 11.80; 9.69; 9.32; 7.84; 7.72. HR-MS (MALDI): 458.2088 ([M+H]^+^, C_24_H_29_FN_3_O_5_^+^; calcd. 458.2091).

*1-Cyclopropyl-6-fluoro-8-methoxy-7-(3-(N-methylbenzamido)piperidin-1-yl)-4-oxo-1,4-dihydroquinoline-3-carboxylic acid* **(12-e)**

Compound **12-e**, 0.22 g was `obtained, light yellow oil, yield 87%. ^1^H-NMR (300 MHz, CDCl_3_): 14.72 (s, 1H); 8.80 (s, 1H); 7.86 (d, *J* = 12.1 Hz, 1H); 7.40 (br, 5H); 4.87 - 4.73 (m, 1H); 4.01 - 3.90 (m, 2H); 3.52 - 3.45 (m, 1H); 3.40 - 3.30 (m, 3H); 3.13 - 3.04 (m, 3H); 1.91 - 1.84 (m, 4H); 1.31- 1.20 (m, 3H); 1.03 (br, 2H); 0.89 - 0.84 (m, 1H). HR-MS (ESI): 494.2089 ([M+H]^+^, for C_27_H_29_FN_3_O_5_^+^; calcd. 494.2091).

**Synthetic experiments of compound 13-e to 18-e**

0.1 g (1.00 eq, 0.90 mmol) compound **13** was dissolved in 20 mL of DMF, 98 μL (1.10 eq, 0.98 mmol) TEA and 90 μL (1.05 eq, 0.94 mmol) of ethyl chloroformate were slowly added dropwise, stirred in an ice-water bath at 0 ℃ for 30 minutes. Then, 20 mL of DMF solution containing 0.2 g (0.58 eq, 0.51 mmol) of parent compound **1e** was added. The solution was stirred for 24 h at room temperature. As the reaction finished, 40 mL deionized water was added, extracted three times with dichloromethane, combined the extracts, concentrated and separated by silica gel column chromatography, afforded 0.2 g target compound, **13-e**, in the yield of 79%.

Compound **14-e** to **18-e** was synthesized in the same way as compound 13-e, in the yields of 70 - 90%.

*1-Cyclopropyl-6-fluoro-8-methoxy-7-(3-(N-methylfuran-2-carboxamido)piperidin-1-yl)-4-oxo-1,4-dihydroquinoline-3-carboxylic acid* **(13-e)**

Compound **13-e**, 0.25 g was obtained, white solid, yield 78%. M.p. 167.5-168.7 ℃. ^1^H-NMR (300 MHz, CDCl_3_): 14.76 (br, 1H); 8.77 (s, 1H); 7.82 (dd, *J* = 12.0, 2.0 Hz, 1H); 7.45 (br, 1H); 7.00 (d, *J* = 3.4 Hz, 1H); 6.48 - 6.47 (m, 1H); 4.18 - 4.11 (m, 1H); 4.07 - 4.00 (m, 1H); 3.81 (s, 3H); 3.37 - 3.30 (m, 1H); 3.16 (s, 3H); 2.95 (br, 1H); 2.88 - 2.87 (m, 2H); 1.91 - 1.89 (m, 2H); 1.30 - 1.25 (m, 2H); 1.23 - 1.21 (m, 2H); 1.01 - 1.00 (m, 2H). ^13^C-NMR (100 MHz, CDCl_3_): 177.06; 166.75; 161.62; 156.62 (d, *J* = 251.8 Hz); 156.43; 149.89; 148.21; 145.30; 139.58; 135.18; 122.14; 116.33; 111.35; 108.14; 107.71; 62.36; 52.79; 51.20; 40.62; 36.49; 29.40; 25.69; 14.72; 9.60; 9.48. HR-MS (MALDI): 484.1884 ([M+H]^+^, C_25_H_27_FN_3_O_6_+; calcd. 484.1884).

*1-Cyclopropyl-6-fluoro-8-methoxy-7-(3-(N-methyltetrahydrofuran-2-carboxamido)piperidin-1-yl)-4-oxo-1,4-dihydroquinoline-3-carboxylic acid* **(14-e)**

Compound **14-e**, 0.25 g was obtained, white solid, yield 83%. M.p. 140.2-141.2 ℃. ^1^H-NMR (300 MHz, CDCl_3_): 14.76 (s, 1H); 8.77 (d, *J* = 2.8 Hz, 1H); 7.81 (d, *J* = 12.1 Hz, 1H); 4.69 - 4.65 (m, 1H); 4.04 - 4.02 (m, 1H); 3.85 (s, 3H); 3.48 - 3.40 (m, 2H); 3.33 - 3.25 (m, 1H); 3.21 - 3.13 (m, 1H); 3.09 - 3.03 (m, 3H); 2.95 (br, 1H); 2.91 - 2.90 (m, 1H); 2.87 (br, 1H); 2.14 - 2.12 (m, 1H); 2.08- 1.98 (m, 2H); 1.91 - 1.89 (br, 3H); 1.78 - 1.73 (br, 1H); 1.31 - 1.18 (m, 3H); 1.01 - 1.00 (m, 2H). ^13^C-NMR (100 MHz, CDCl_3_): 177.05; 171.71; 166.70; 156.70 (d, *J* = 251.8 Hz); 149.91; 146.05; 139.52; 133.76; 122.24; 108.11; 107.65; 76.16; 69.19; 62.46; 53.71; 52.92; 51.26; 40.65; 36.49; 30.78; 28.60; 27.36; 25.78; 9.70; 9.38. HR-MS (MALDI): 488.2196 ([M+H]^+^, C_25_H_31_FN_3_O_6_^+^; calcd. 488.2197).

*1-Cyclopropyl-6-fluoro-8-methoxy-7-(3-(N-methylnicotinamido)piperidin-1-yl)-4-oxo-1,4-dihydroquinoline-3-carboxylic acid* **(15-e)**

Compound **15-e**, 0.25 g was obtained, light yellow solid, yield 83%. ^1^H-NMR (300 MHz, CDCl_3_); 14.78 (br, 1H); 8.77 (d, *J* = 4.3 Hz, 1H); 8.58 - 8.43 (m, 1H); 7.85 – 7.81 (m, 1H); 7.79 - 7.77 (m, 1H); 4.11 - 3.89 (m, 1H); 3.87 (br, 1H); 3.56 - 3.33 (m, 3H); 3.10 - 3.01 (m, 3H); 2.12 - 2.00 (m, 2H); 2.12 - 2.01 (m, 2H); 1.86 - 1.77 (m, 2H); 1.47 - 1.42 (m, 1H); 1.29 - 1.18 (m, 4H); 1.03 - 0.99 (m, 2H); 0.87 - 0.84 (m, 1H). ^13^C-NMR (100 MHz, CDCl_3_): 177.05; 166.73; 162.54; 156.62 (d, *J* = 252.6 Hz); 154.75; 149.87; 148.25; 145.98; 137.10; 133.83; 129.89; 124.44; 123.52; 122.19; 108.09; 107.66; 62.18; 53.57; 50.97; 40.63; 31.66; 28.09; 25.60; 14.23; 9.64; 9.39. HR-MS (ESI): 495.2042 ([M+H]^+^, C_26_H_28_FN_4_O_5_^+^; calcd. 495.2044).

*1-Cyclopropyl-6-fluoro-8-methoxy-7-(3-(N-methylcyclopentanecarboxamido)piperidin-1-yl)-4-oxo-1,4-dihydroquinoline-3-carboxylic acid* **(16-e)**

Compound **16-e**, 0.35 g was obtained, light yellow solid, yield 72%. M.p. 105.1-106.4℃. ^1^H-NMR (300 MHz, CDCl_3_): 14.75 (s, 1H); 8.78 (s, 1H); 7.85 - 7.79 (m, 1H); 4.78 - 4.70 (m, 1H); 4.04 (br, 1H); 3.86 - 3.79 (m, 3H); 3.49 - 3.39 (m, 2H); 3.26 - 3.19 (m, 1H); 3.01 - 2.96 (m, 3H); 2.90 - 2.89 (m, 1H); 1.98 (br, 1H); 1.87 -1.75 (m, 8H); 1.60 - 1.56 (m, 2H); 1.30 - 1.18 (m, 4H); 1.01 (br, 2H). ^13^C-NMR (100 MHz, CDCl_3_): 176.80; 166.77; 156.79 (d, *J* = 252.1 Hz); 155.54; 149.86; 146.13; 139.61; 133.82; 122.16; 108.20; 107.68; 62.01; 53.99; 52.963; 51.13; 41.13; 30.90; 30.49; 30.18; 30.12; 29.98; 28.54; 25.88; 14.72; 9.63; 9.30. HR-MS (MALDI): 486.2403 ([M+H]^+^, C_26_H_33_FN_3_O_5_^+^; calcd. 486.2404).

**Synthesis experiment of compound 19-e, 20-e**

0.2 g (1 eq, 0.36 mmol) **17-e** was dissolved and stirred in 10 mL of TFA and dichloromethane (v/v=1:1) for about 5 h to remove the Boc protection group. Then excess TFA was removed under vacuum. The de-protected product was washed with cold diethyl ether, filtered and dried under vacuum afforded 0.15 g **19-e**, in the yields of 91%. Compound **20-e** was obtained similarly.

Compound **20-e** was synthesized in the same way as compound **19-e**, in the yields of 91%.

*7-(3-(2-Amino-N-methylpropanamido)piperidin-1-yl)-1-cyclopropyl-6-fluoro-8-methoxy-4-oxo-1,4-dihydroquinoline-3-carboxylic acid* **(19-e)**

Compound **19-e**, 0.10 g was obtained, dark yellow oil, yield 81%. ^1^H-NMR (300 MHz, CD_3_OD): 8.72 (br, 1H); 7.57 (dd, *J* = 12.2, 4.4 Hz, 1H); 4.55 - 4.55 (m, 1H); 4.37 - 4.33 (m, 1H); 3.79 - 3.75 (m, 3H); 3.48 - 3.41 (m, 2H); 2.93 - 2.91 (m, 3H); 2.84 (br, 1H); 2.80 - 2.79 (m, 2H); 1.85 (br, 3H); 1.46 - 1.39 (m, 2H); 1.25 - 1.19 (m, 2H); 1.18 - 1.14 (m, 2H); 1.00 (br, 2H). ^13^C-NMR (100 MHz, CD_3_OD): 178.26; 170.26; 164.85; 160.52 (d, *J* = 260.4 Hz); 151.80; 147.58; 141.16; 135.56; 112.89; 116.04; 108.03; 62.03; 54.42; 53.51; 52.18; 42.13; 36.94; 31.65; 30.82; 26.79; 16.30; 10.03; 9.79. HR-MS (ESI): 461.2193 ([M+H]^+^, C_23_H_30_FN_4_O_5_^+^; calcd. 461.2200).

*7-(3-(2-Amino-N-methylacetamido)piperidin-1-yl)-1-cyclopropyl-6-fluoro-8-methoxy-4-oxo-1,4-dihydroquinoline-3-carboxylic acid* **(20-e)**

Compound **20-e**, 0.11 g was obtained, yellow oil, yield 91%. ^1^H-NMR (300 MHz, CD_3_OD): 8.73 (d, *J* = 2.8 Hz, 1H); 7.56 (t, *J* = 13.2 Hz, 1H); 4.13 - 4.10 (m, 1H); 3.90 (br, 1H); 3.80 - 3.79 (m, 3H); 3.49 - 3.42 (m, 2H); 3.35 - 3.31 (m, 1H); 3.14 (br, 1H); 2.96 - 2.94 (m, 3H); 1.89 (br, 1H); 1.87 - 1.82 (m, 2H); 1.26 - 1.25 (m, 1H); 1.23 - 1.19 (m, 2H); 1.17 - 1.10 (m, 2H); 1.02 - 1.00 (m, 2H). ^13^C-NMR (100 MHz, CD_3_OD): 178.27; 168.26; 164.38; 157.78 (d, *J* = 250.7 Hz); 151.81; 147.49; 141.13; 122.86; 135.60; 117.46; 108.04; 63.48; 54.50; 53.30; 51.97; 42.10; 41.50; 30.07; 29.79; 29.15; 28.72; 28.16; 26.69; 10.07; 9.88. HR-MS (ESI): 447.2041 ([M+H]^+^, C_22_H_28_FN_4_O_5_^+^; calcd. 447.2044).

**Synthetic experiments of compound 21-e**

The 0.2 g (1.00 eq, 0.51 mmol) parent compound **1e** was dissolved in 10 mL of formic acid solution and 200 μL (3.00 eq, 1.53 mmol) of formaldehyde solution was slowly added dropwise, heated and stirred at 110 °C for 24 h. After completion of the reaction, 20 - 40 mL of deionized water was added to the reaction solution, extracted three times with dichloromethane. Combined with the extracts, concentrated and cold diethyl ether was added, 0.17 g target compound **21-e** was obtained as light yellow oil, the yield was 82%.

*1-Cyclopropyl-7-(3-(dimethylamino)piperidin-1-yl)-6-fluoro-8-methoxy-4-oxo-1,4-dihydroquinoline-3-carboxylic acid* **(21-e)**

Compound **21-e**, 0.17 g was obtained, light yellow oil, yield 82%. ^1^H-NMR (300 MHz, CDCl_3_): 14.79 (s, 1H); 8.78 (s, 1H); 7.82 (t, *J* = 12.1 Hz, 1H); 4.07 - 4.00 (m, 1H); 3.75 (s, 3H); 3.70 (br, 1H); 3.51 - 3.44 (m, 1H); 3.15 - 2.99 (m, 2H); 2.54 - 2.47 (m, 1H); 2.17 (brs, 6H); 1.92 - 1.86 (m, 1H); 1.80 - 1.68 (m, 1H); 1.50 -1.38 (m, 1H); 1.28 - 1.15 (m, 3H); 1.04 - 0.95 (m, 2H). ^13^C-NMR (100 MHz, CDCl_3_): 206.92; 177.05; 166.82; 156.32 (d, *J* = 251.3 Hz); 149.81; 145.37; 140.21; 133.99; 121.51; 107.95; 62.46; 61.74; 54.47; 51.31; 42.15; 40.58; 30.92; 27.49; 25.52; 9.68; 9.41. HR-MS (ESI): 404.1983 ([M+H]^+^, C_21_H_27_FN_3_O_4_^+^; calcd. 404.1986).

**Synthesis experiment of compound 22-e**

1.10 g (1.00 eq, 12.90 mmol) of cyanoacetic acid was dissolved in anhydrous dichloromethane, stirred at -10 ℃ for 10 min, then 1.04 mL of oxalyl chloride and a catalytic amount of DMF were slowly added. After the reaction is completed, the excess oxalyl chloride and solvent were removed. Then a solution of 0.39 g **1e** in 20 mL DMF was added and reacted at room temperature for 12 h. After that, 20 - 40 mL of deionized water was added, extracted three times with dichloromethane. Combined with the extracts and evaporated, in which the cold diethyl ether was added, precipitation occurred, filtration and drying afforded 0.10 g target compound **22-e** as light red powder, in the yield of 22%.

*7-(3-(2-Cyano-N-methylacetamido)piperidin-1-yl)-1-cyclopropyl-6-fluoro-8-methoxy-4-oxo-1,4-dihydroquinoline-3-carboxylic acid* **(22-e)**

Compound **22-e**, 0.10 g was obtained, light red powder, yield 22%. M.p. 184.8-186.2℃. ^1^H-NMR (300 MHz, CDCl_3_): 14.75 (brs, 1H); 8.82 (d, *J* = 3.2 Hz, 1H); 7.88 (d, *J* = 12.0 Hz, 1H); 4.16 - 4.09 (m, 1H); 3.86 (s, 2H); 3.53 (br, 1H); 3.52 (br, 1H); 3.50 (s, 3H); 3.48 (s, 3H); 3.45 (br, 1H); 3.04 (br, 2H); 1.27 - 1.26 (m, 4H); 1.25 (br, 2H); 1.20 (br, 2H). HR-MS (ESI): 457.1878 ([M+H]^+^, C_23_H_26_FN_4_O_5_^+^, calcd. 457.1887).

**Synthetic experiments of compound e-1 and f-1**

0.39 g (1.00 eq, 1.00 mmol) **1e** was dissolved in 10 mL of water-free DMF, 150 μL (1.50 eq, 1.50 mmol) TEA and 169 μL (2.20 eq, 2.20 mmol) of chloroacetyl chloride were added in sequence. This solution was stirred at room temperature for 24 h, as the reaction was completed, 30 mL of deionized water was added to precipitate the reaction. Filtration and the filter cake were washed with deionized water. Then drying, 0.41 g of the intermediate, **2e**, was obtained, in a yield of 88%. 0.10 g (1.00 eq, 0.22 mmol) **2e** was dissolved in 10 mL acetonitrile, and 22 mg (1.20 eq, 0.26mmol) sodium triazole was added to the solution, stirred at 50 ℃ for 48 h. As the reaction was completed, the reaction solution was concentrated and passed through a silica gel column to give 0.07 g of the title compound **e-1**, in a yield of 58%. Compound **f-1** was synthesized in the same way as compound **e-1** in the yield of 50 - 80%.

*1-Cyclopropyl-6-fluoro-8-methoxy-7-(3-(N-methyl-2-(1H-1,2,4-triazol-1-yl)acetamido) piperidin-1-yl)-4-oxo-1,4-dihydroquinoline-3-carboxylic acid* **(e-1)**

Compound **e-1**, 0.07 g was obtained, light yellow oil, yield 58%. ^1^H-NMR (300 MHz, CDCl_3_): 14.87 (s, 1H); 8.81 (d, *J* = 5.6 Hz, 1H); 8.27 (d, *J* = 12.6 Hz, 1H); 8.20 (br, 1H); 7.92 - 7.81 (m, 1H); 4.69 - 4.64 (m, 2H); 4.06 - 4.01 (m, 1H); 3.82 (br, 3H); 3.54 - 3.45 (m, 2H); 3.36 (br, 1H); 3.30 - 3.23 (m, 1H); 3.09 (s, 3H); 2.99 (br, 1H); 1.38 (br, 2H); 1.34 (br, 1H); 1.29 (br, 3H); 0.89 (br, 2H). HR-MS (ESI): 499.2102 ([M+H]^+^, C_24_H_28_FN_6_O_5_^+^; calcd. 499.2105).

*7-(1-(2-(1H-1,2,4-Triazol-1-yl)acetyl)octahydro-6H-pyrrolo[3,4-b]pyridin-6-yl)-1-cyclopropyl-6-fluoro-8-methoxy-4-oxo-1,4-dihydroquinoline-3-carboxylic acid* **(f-1)**

Compound **f-1**, 0.08 g was obtained, light yellow oil, yield 72%. ^1^H-NMR (300 MHz, CDCl_3_): 15.03 (br, 1H); 8.75 (s, 1H); 8.29 (s, 1H); 7.99 (s, 1H); 7.75 (d, *J* = 13.8Hz, 1H); 5.18 (br. 2H); 4.66 - 4.56 (m, 1H); 3.88 - 3.81 (m, 2H); 3.58 (s, 3H); 3.53 (br, 1H); 3.37 - 3.29 (m, 2H); 2.47 - 2.32 (m, 2H); 1.96 - 1.93 (m, 2H); 1.68 - 1.61 (m, 2H); 1.16 - 1.10 (m, 2H); 0.94 - 0.82 (m, 2H). HR-MS (ESI): 509.1950 ([M-H]^-^, C_25_H_26_FN_6_O_5_^-^; calcd. 509.1949).

Some Representative Spectra


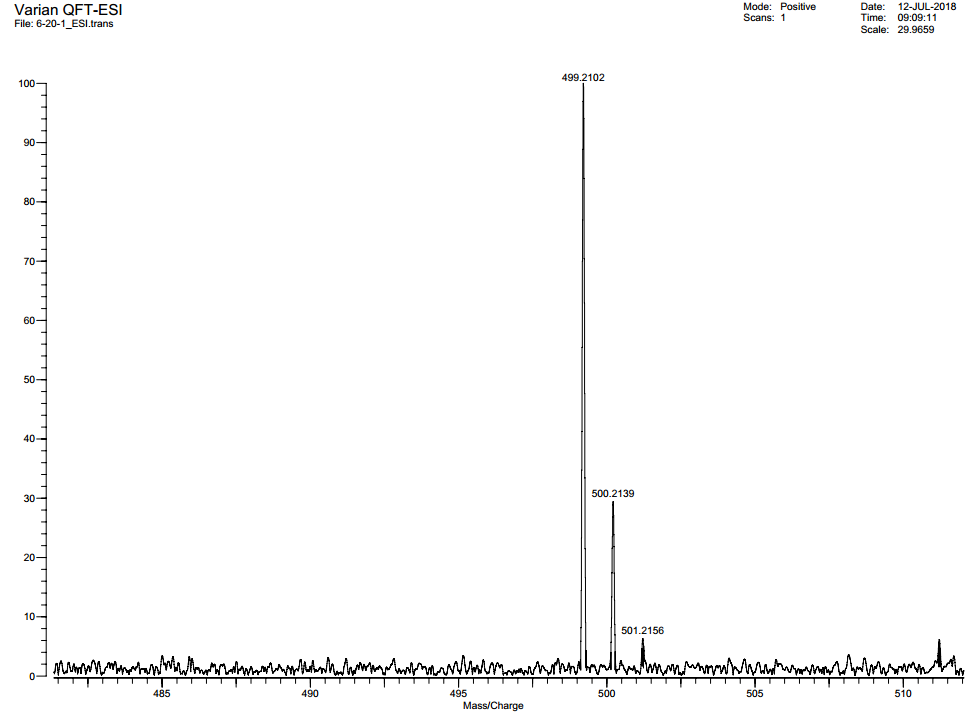


HRMS Spectrum of Compound **e-1**


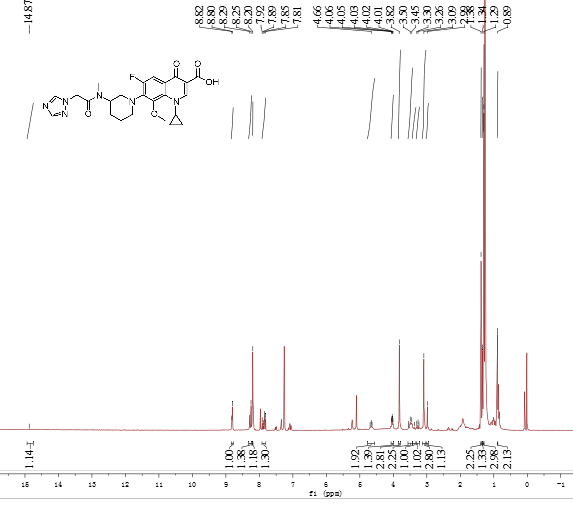


^1^H NMR Spectrum of Compound **e-1**


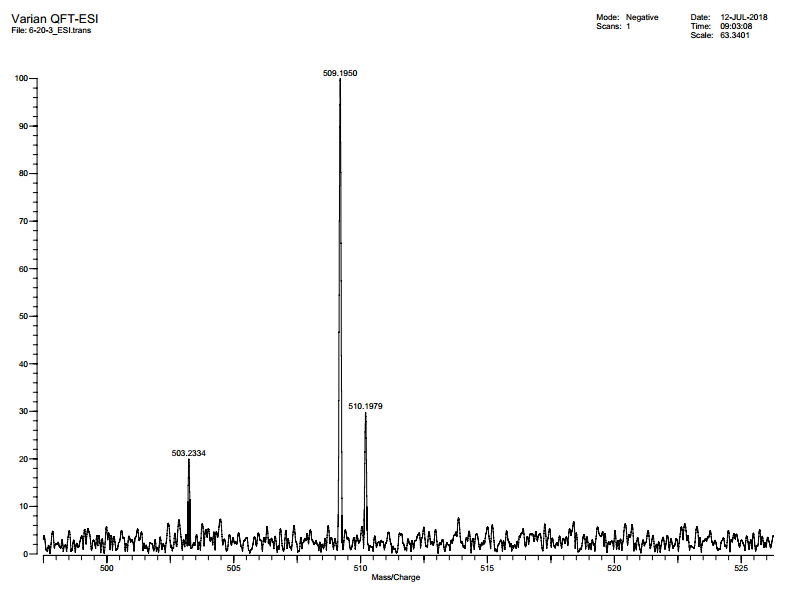


HRMS Spectrum of Compound **f-1**


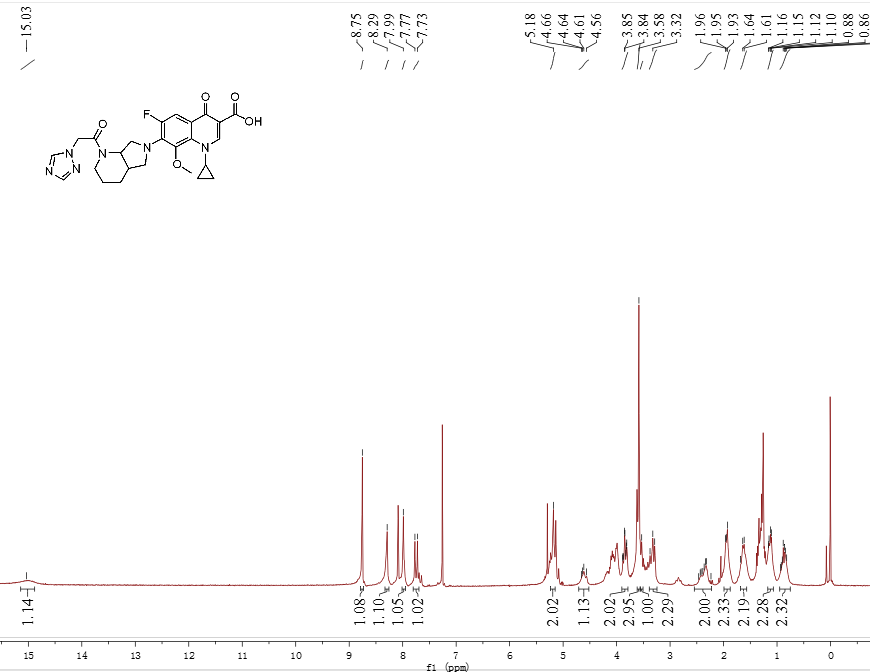


^1^H NMR Spectrum of Compound **f-1**


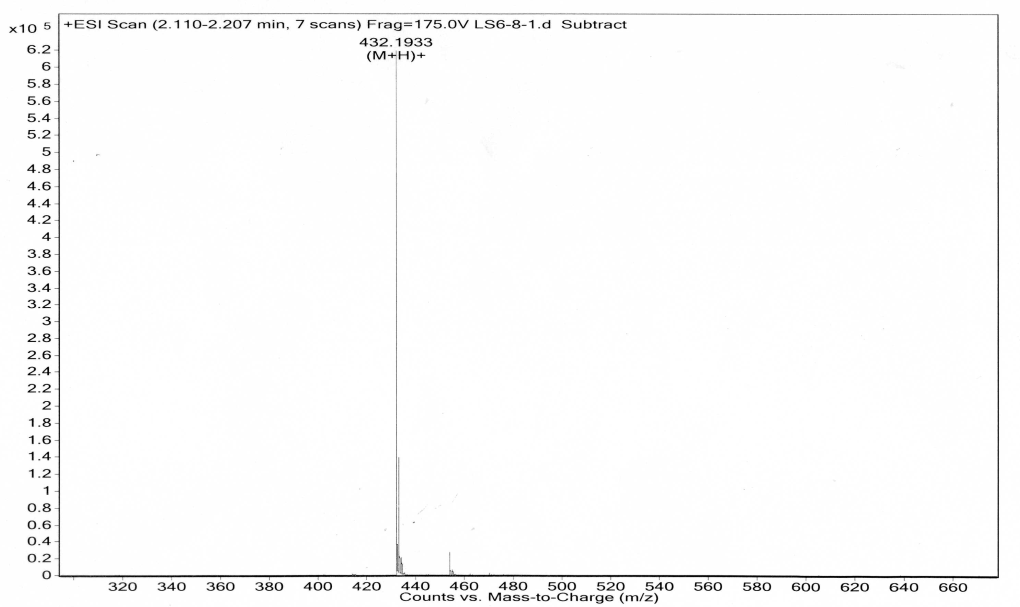


HRMS Spectrum of Compound **2-e**


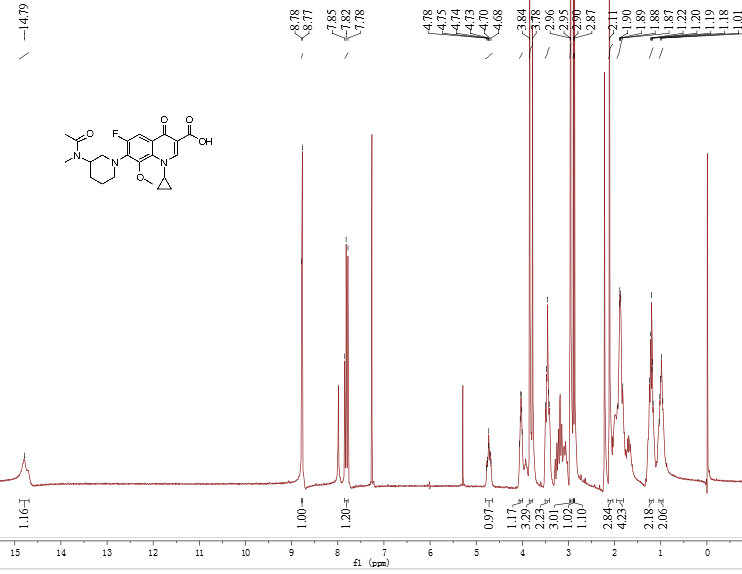


^1^H NMR Spectrum of Compound **2-e**


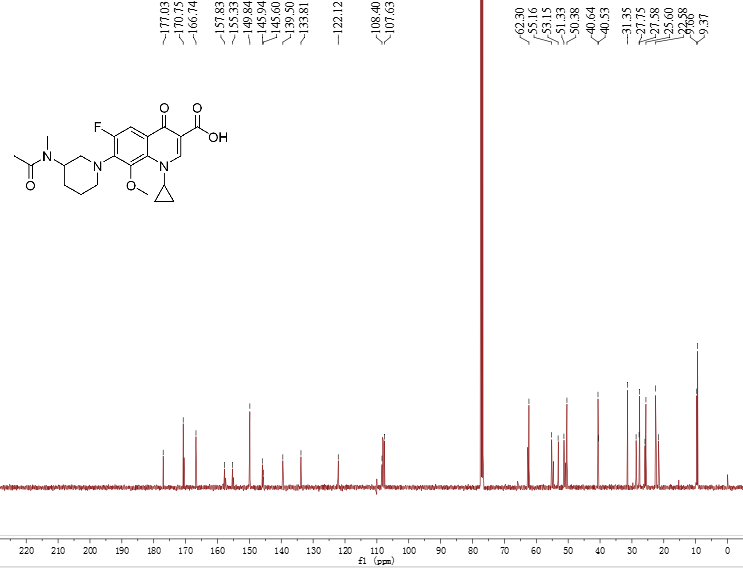


^13^C NMR Spectrum of Compound **2-e**


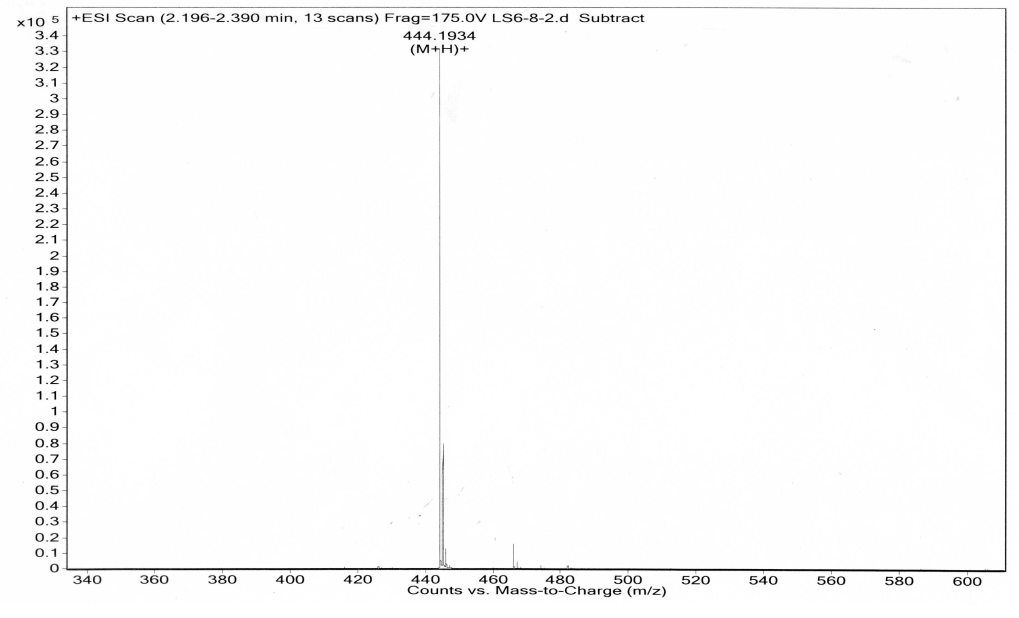


HRMS Spectrum of Compound **2-f**


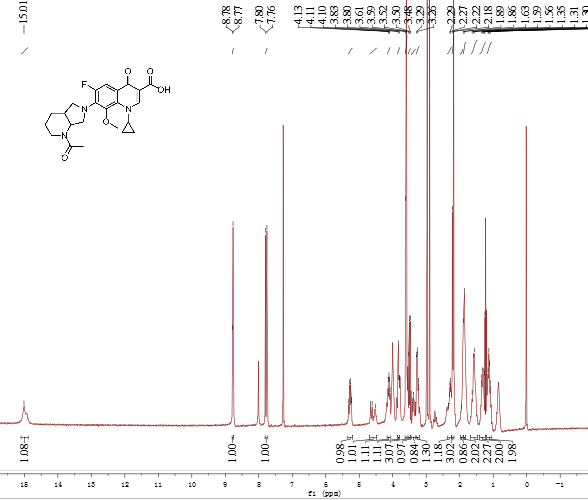


^1^H NMR Spectrum of Compound **2-f**


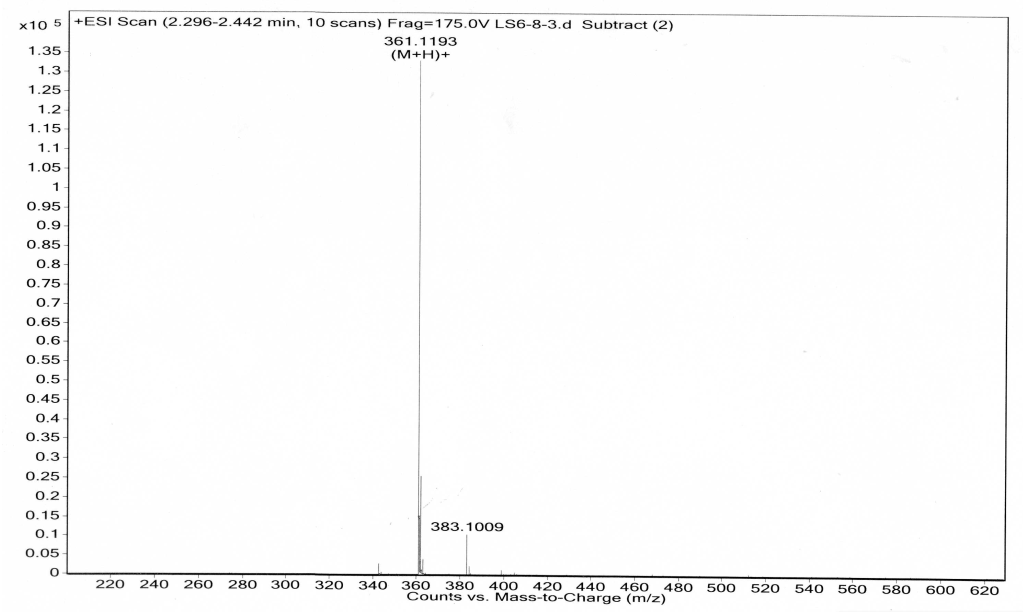


HRMS Spectrum of Compound **2-g**


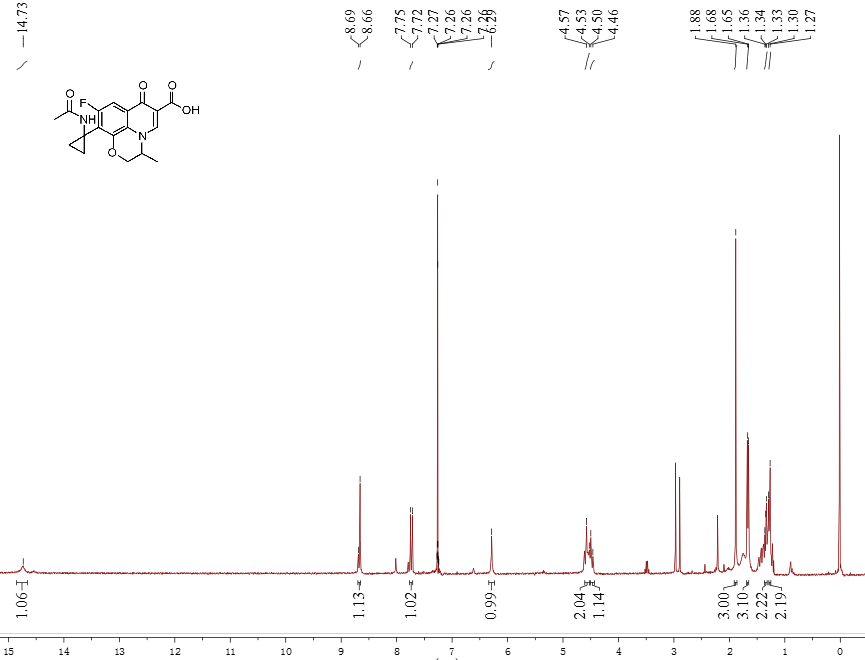


^1^H NMR Spectrum of Compound **2-g**


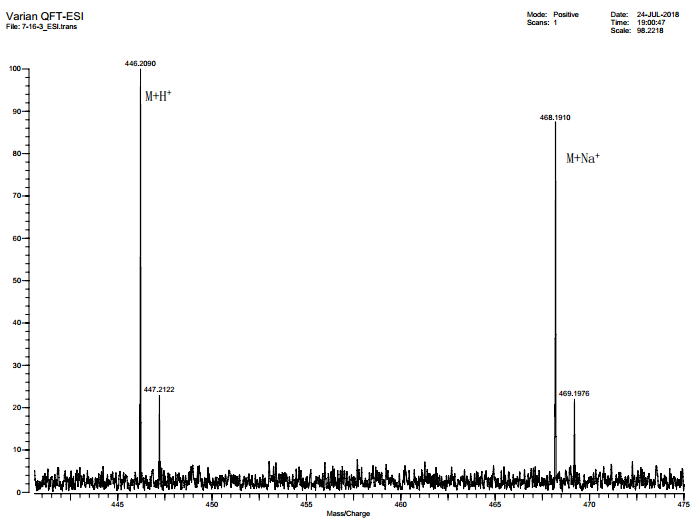


HRMS Spectrum of Compound **3-e**


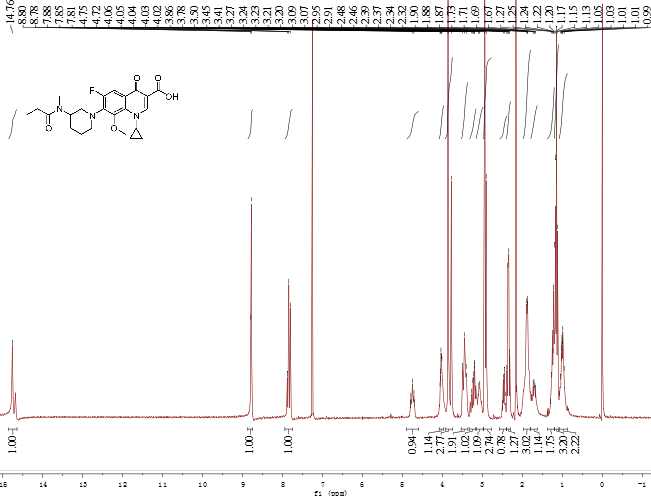


^1^H NMR Spectrum of Compound **3-e**


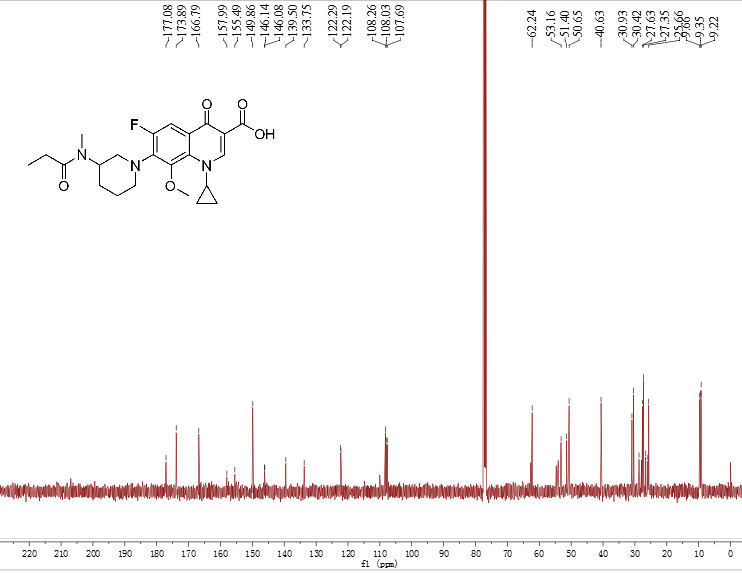


^13^C NMR Spectrum of Compound **3-e**

**
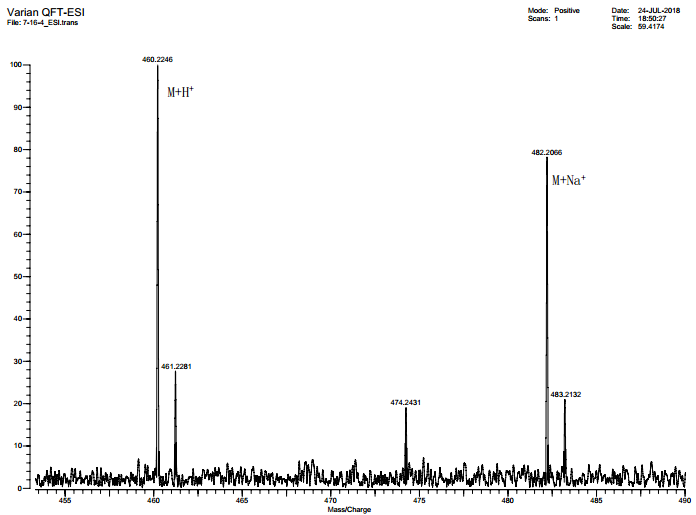
**

HRMS Spectrum of Compound **4-e**

^
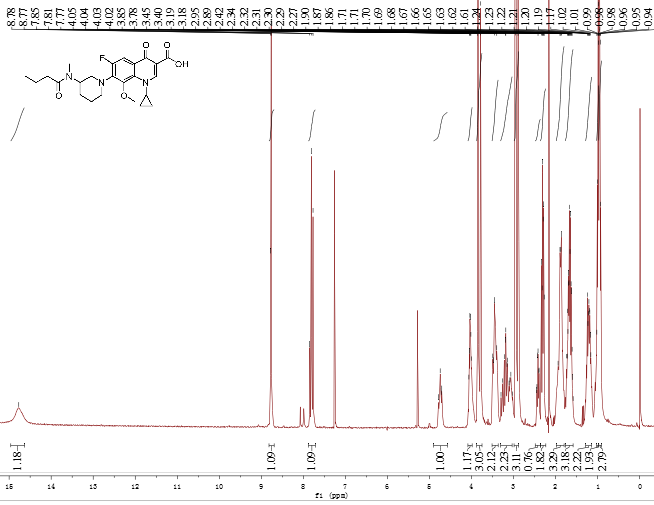
^

^1^H NMR Spectrum of Compound **4-e**


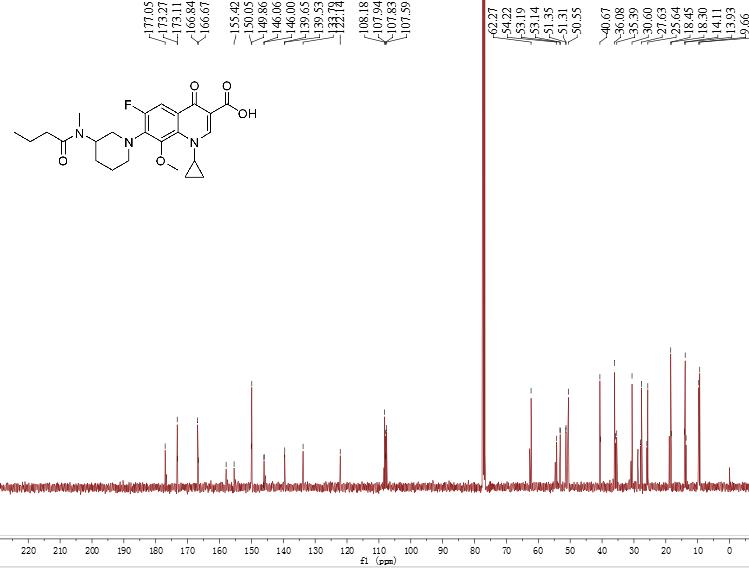


^13^C NMR Spectrum of Compound **4-e**
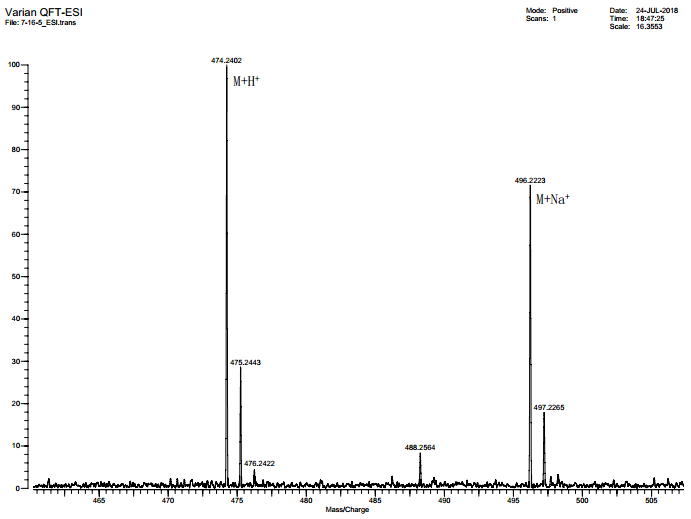


HRMS Spectrum of Compound **5-e**


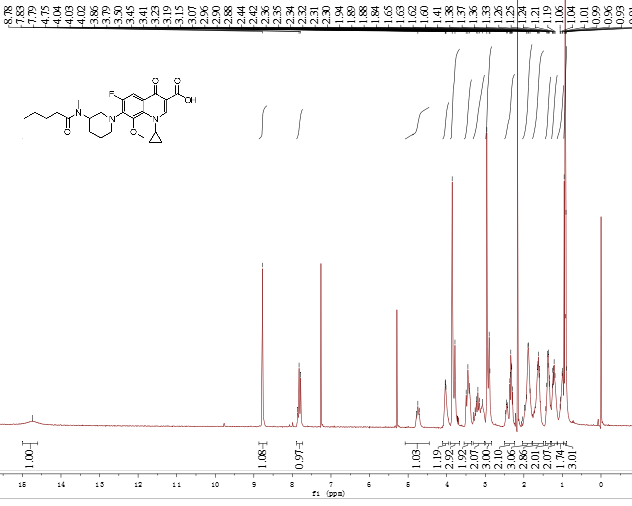


^1^H NMR Spectrum of Compound **5-e**


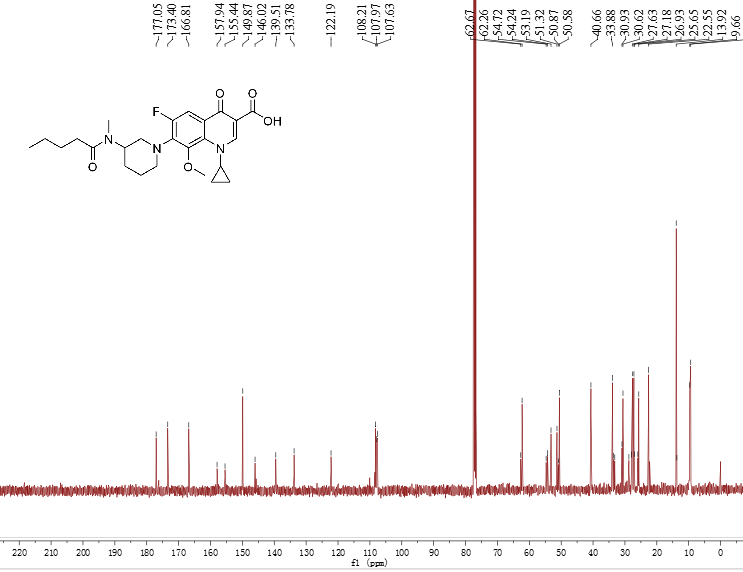


^13^C NMR Spectrum of Compound **5-e**


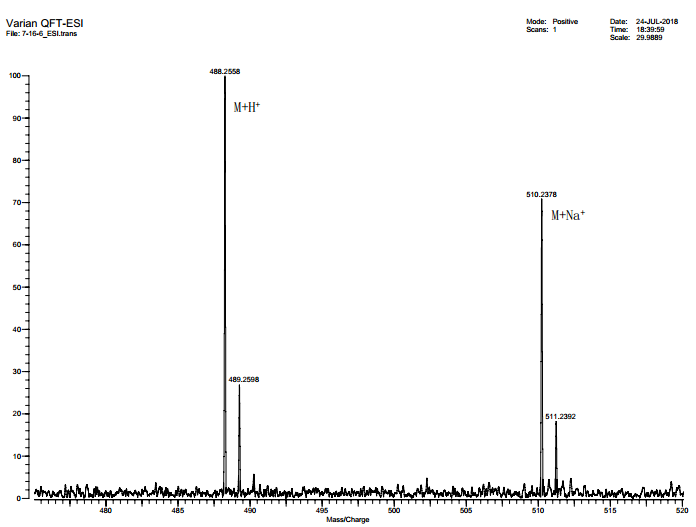


HRMS Spectrum of Compound **6-e**


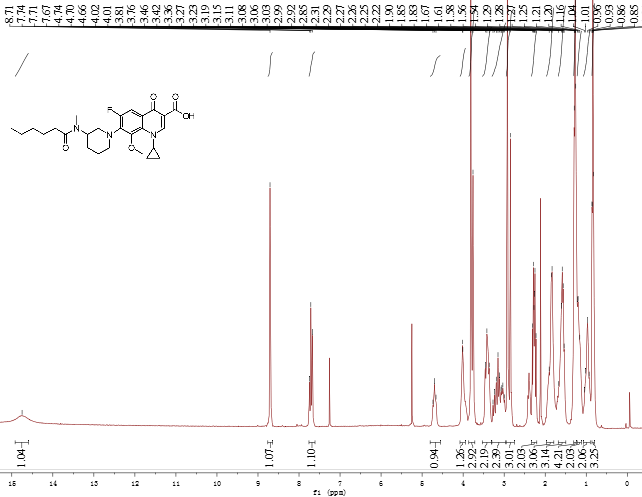


^1^H NMR Spectrum of Compound **6-e**


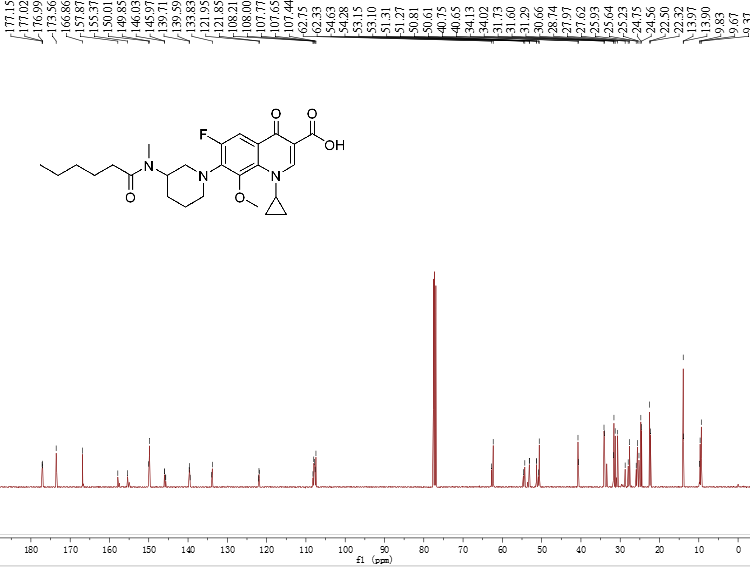


^13^C NMR Spectrum of Compound **6-e**

**
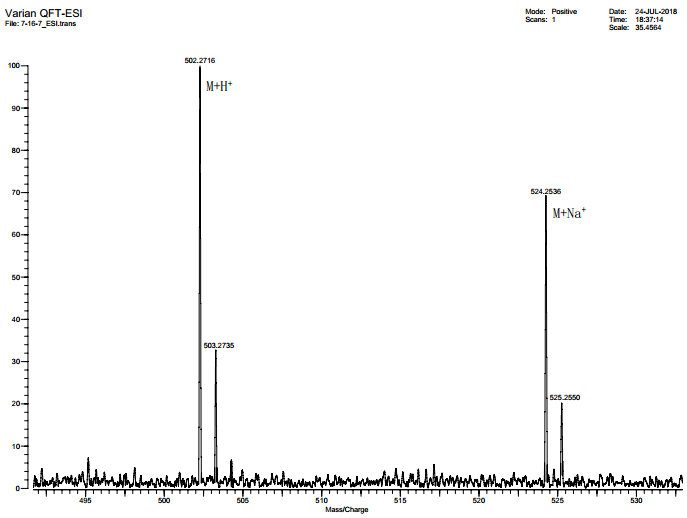
**

HRMS Spectrum of Compound **7-e**

**
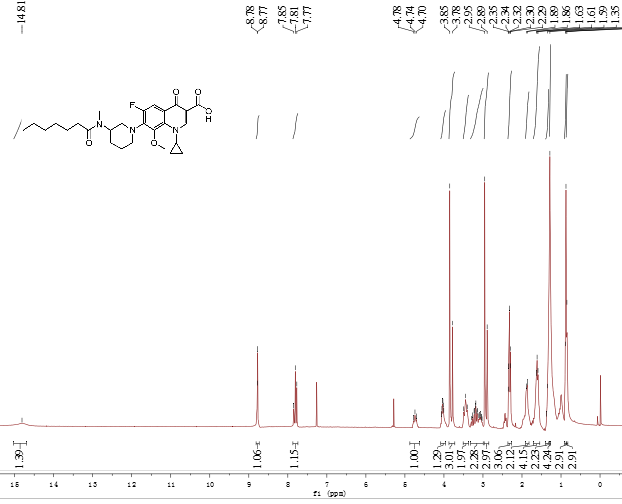
**

^1^H NMR Spectrum of Compound **7-e**


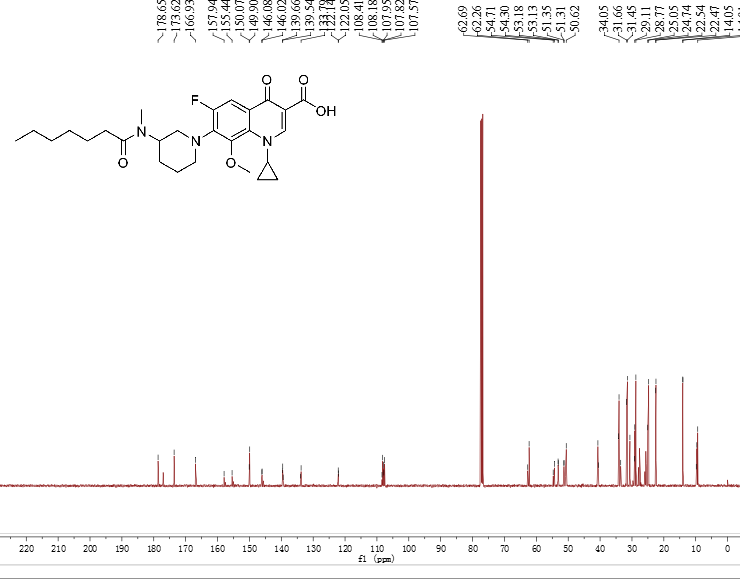


^13^ C NMR Spectrum of Compound **7-e**


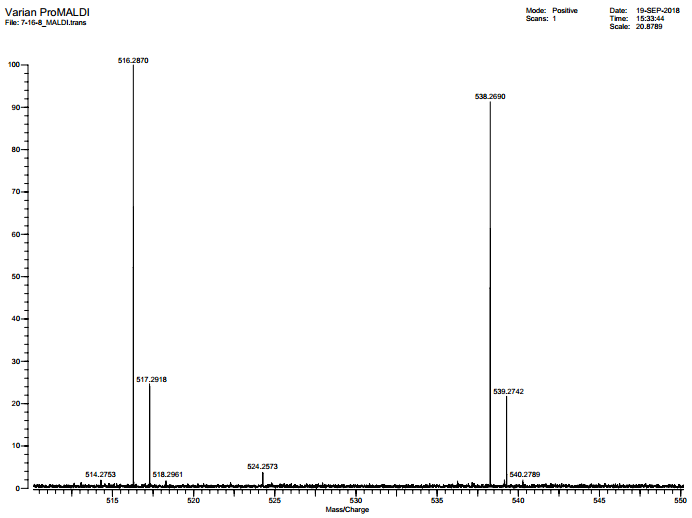


HRMS Spectrum of Compound **8-e**

**
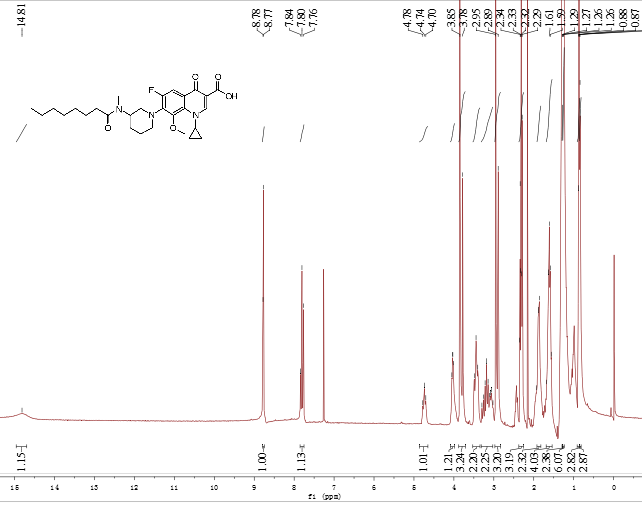
**

^1^H NMR Spectrum of Compound **8-e**


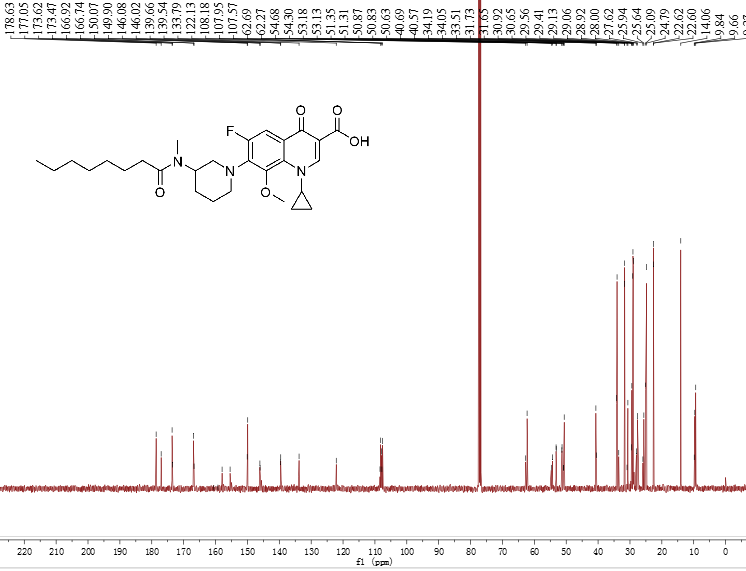


^13^C NMR Spectrum of Compound **8-e**


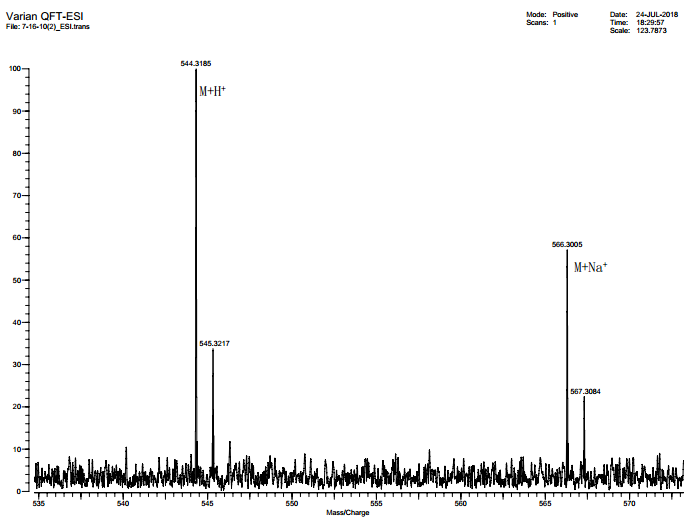


HRMS Spectrum of Compound **10-e**

**
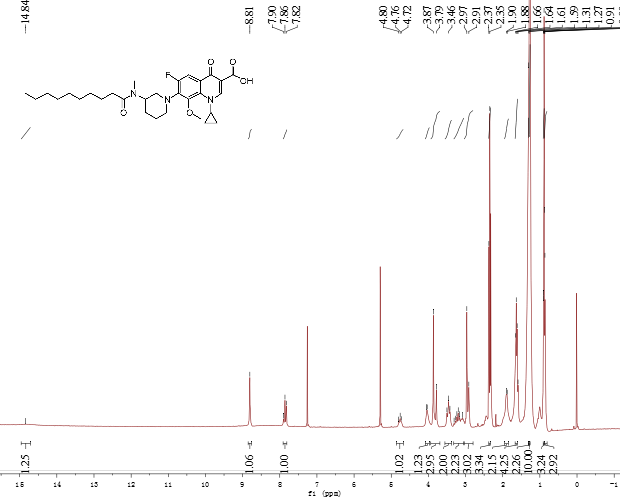
**

^1^H NMR Spectrum of Compound **10-e**

**
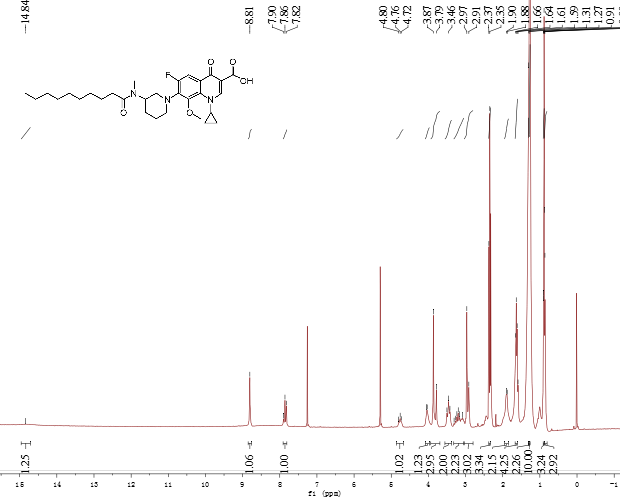
**

^13^C NMR Spectrum of Compound **10-e**

^
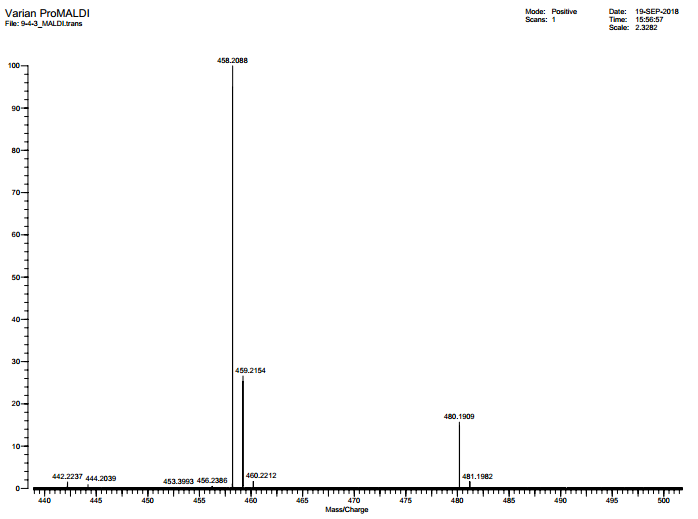
^

HRMS Spectrum of Compound **11-e**

^
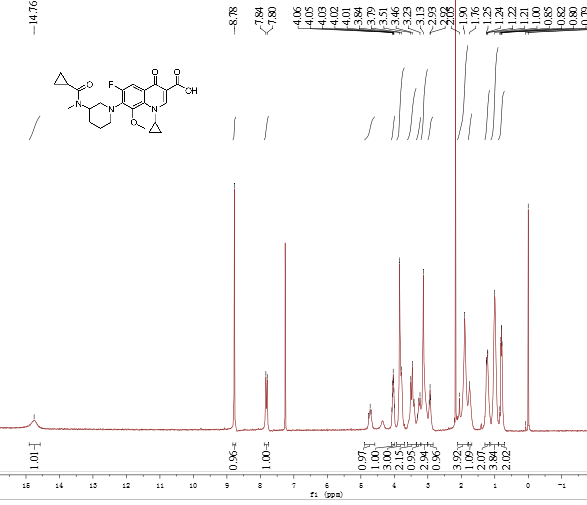
^

^1^H NMR Spectrum of Compound **11-e**


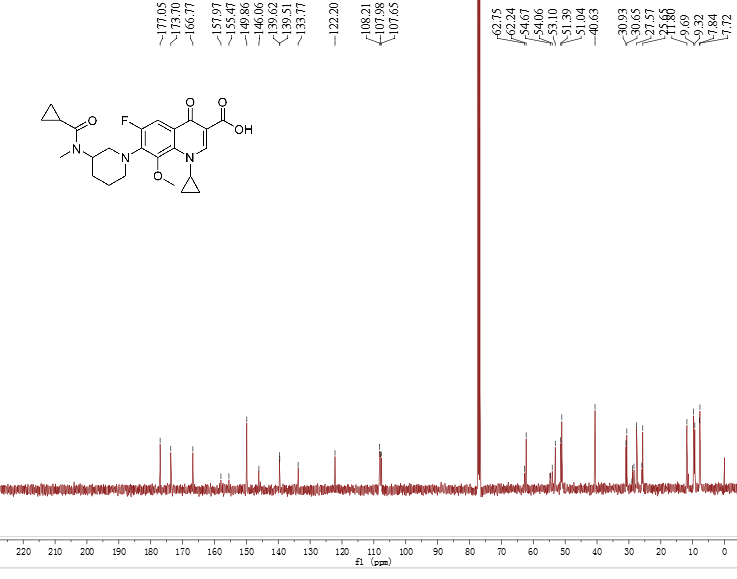


^13^C NMR Spectrum of Compound **11-e**


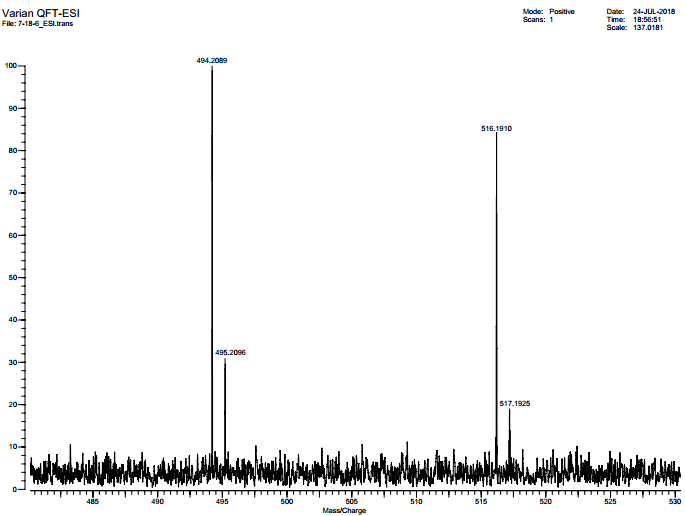


HRMS Spectrum of Compound **12-e**


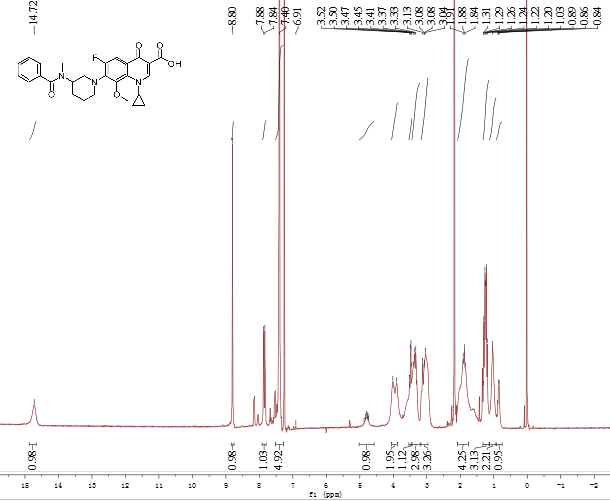


^1^H NMR Spectrum of Compound **12-e**


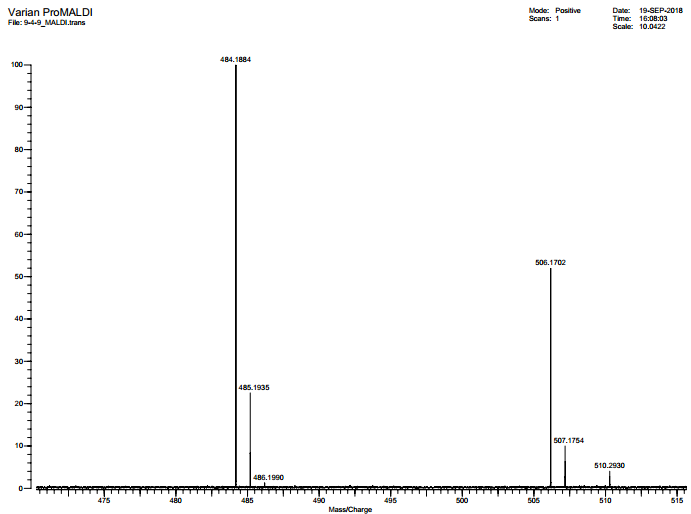


HRMS Spectrum of Compound **13-e**

**
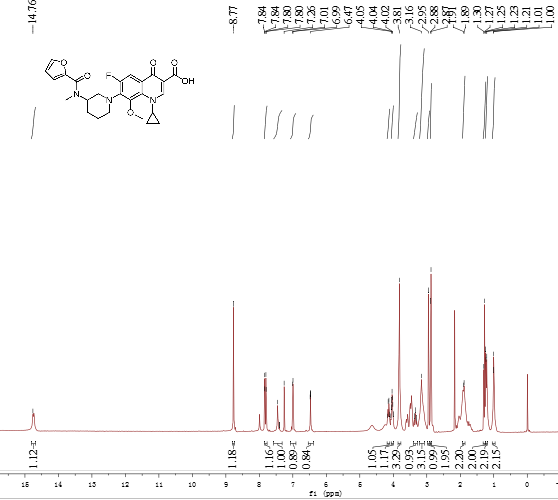
**

^1^H NMR Spectrum of Compound **13-e**


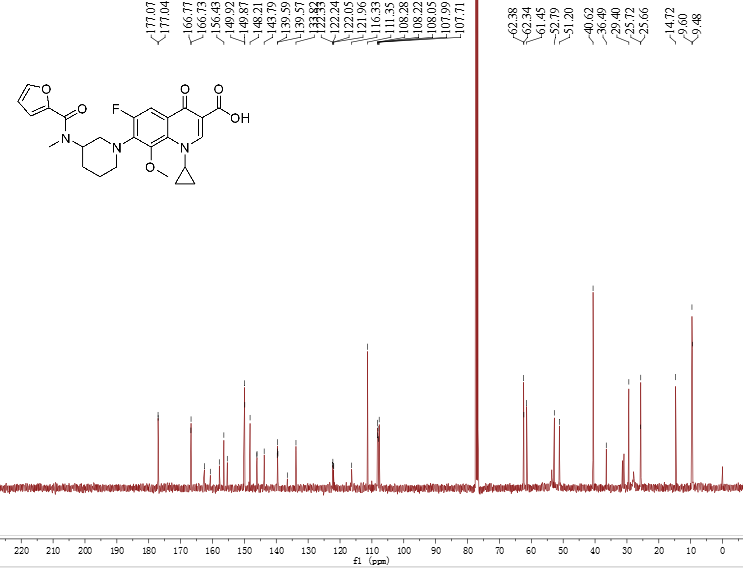


^13^C NMR Spectrum of Compound **13-e**


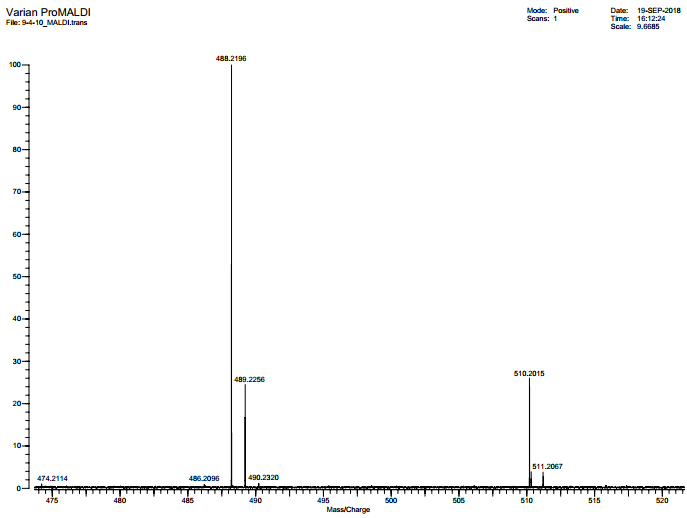


HRMS Spectrum of Compound **14-e**

**
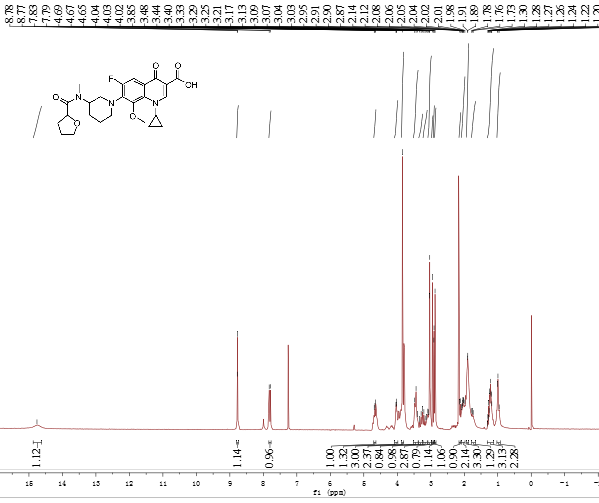
**

^1^H NMR Spectrum of Compound **14-e**


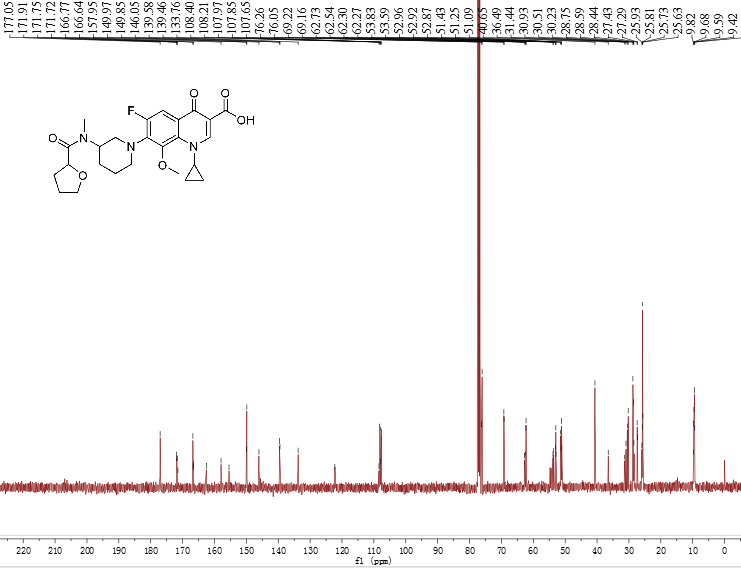


^13^C NMR Spectrum of Compound **14-e**


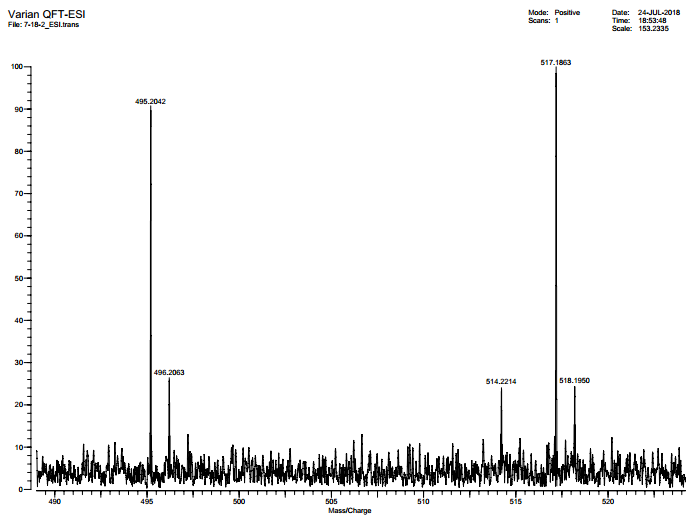


HRMS Spectrum of Compound **15-e**

**
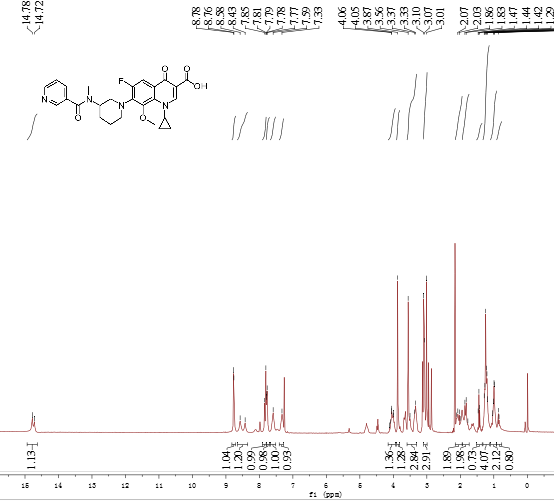
**

^1^H NMR Spectrum of Compound **15-e**


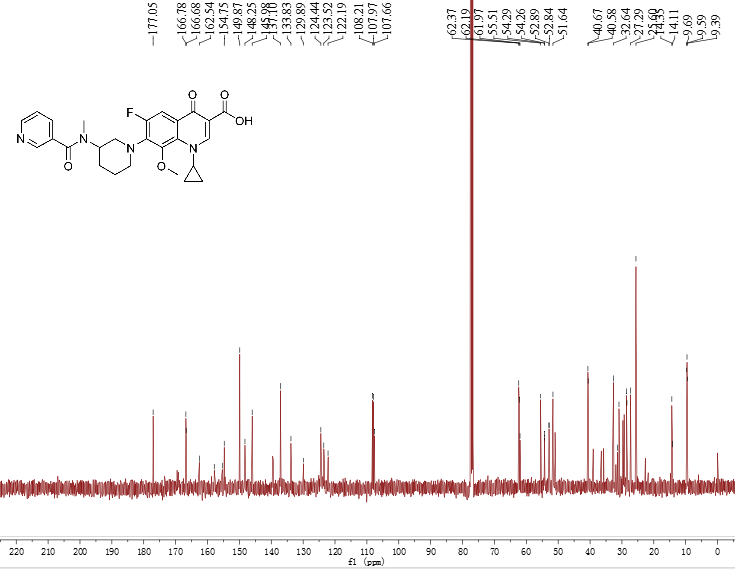


^13^C NMR Spectrum of Compound **15-e**


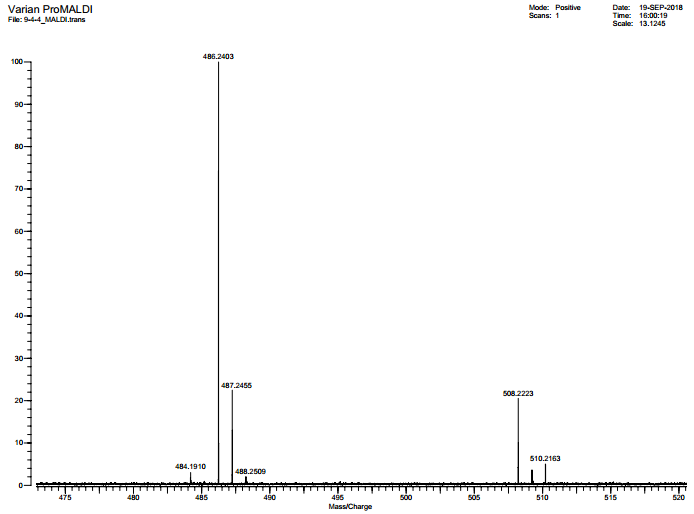


HRMS Spectrum of Compound **16-e**


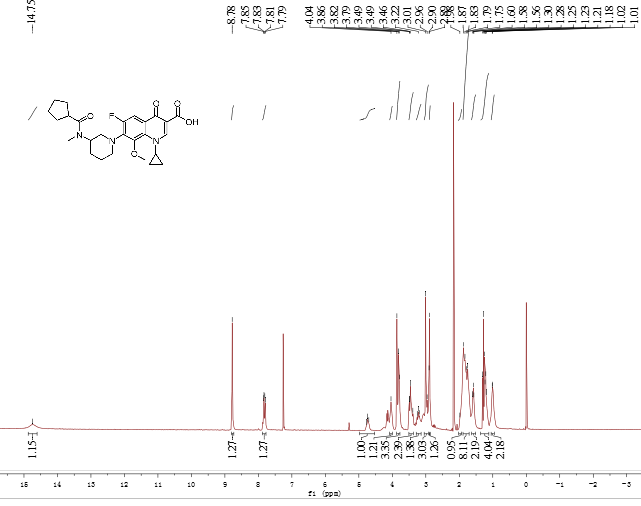


^1^H NMR Spectrum of Compound **16-e**


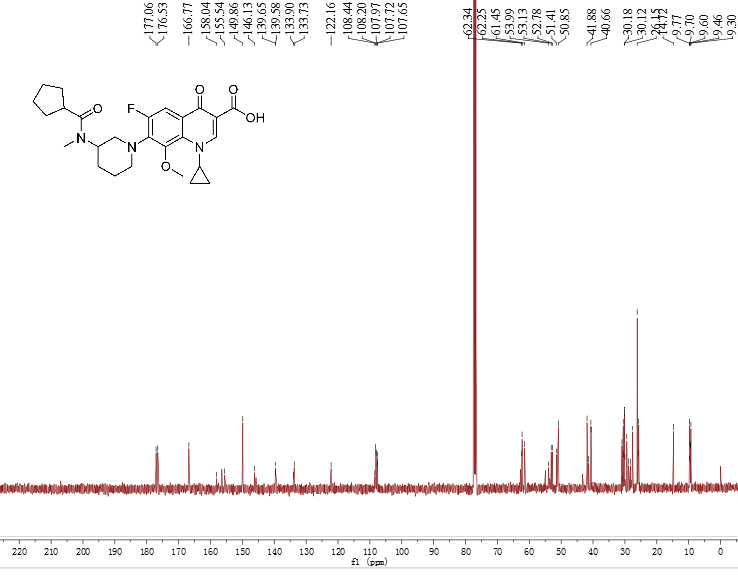


^13^C NMR Spectrum of Compound **16-e**


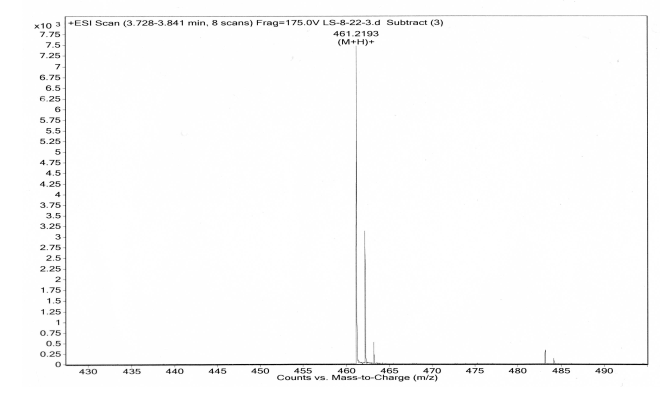


HRMS Spectrum of Compound **19-e**

**
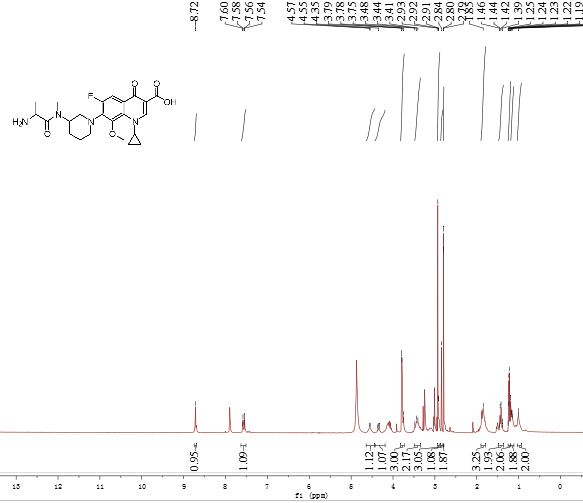
**

^1^H NMR Spectrum of Compound **19-e**


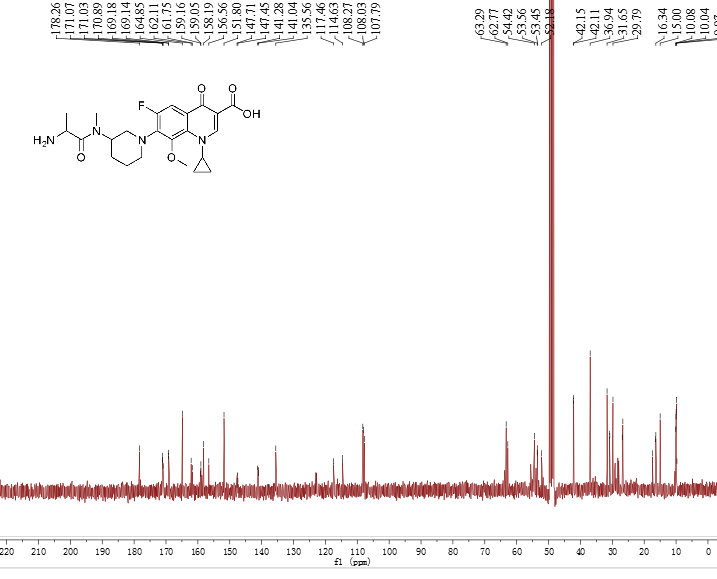


^13^C NMR Spectrum of Compound **19-e**


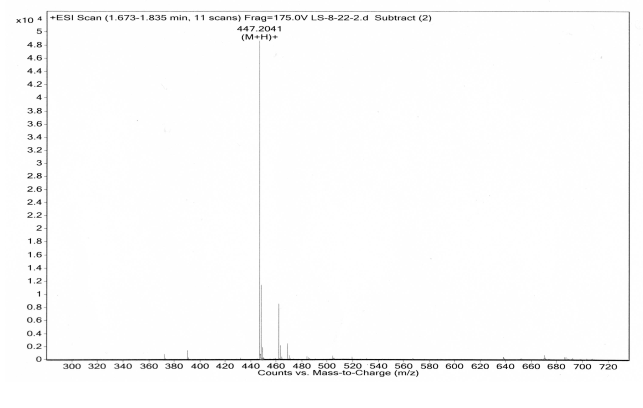


HRMS Spectrum of Compound **20-e**

**
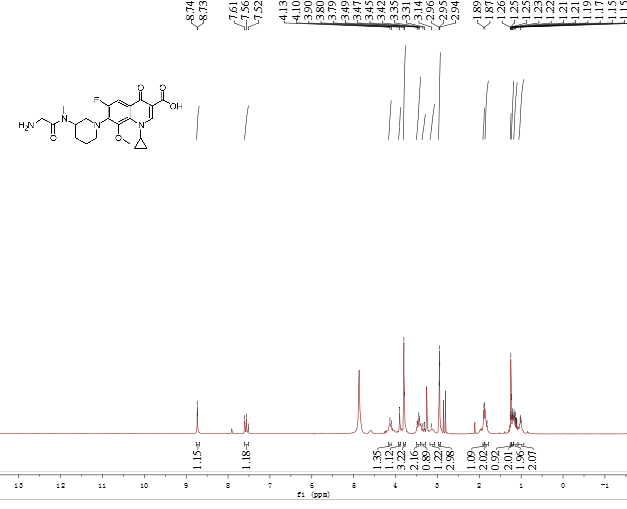
**

^1^H NMR Spectrum of Compound **20-e**


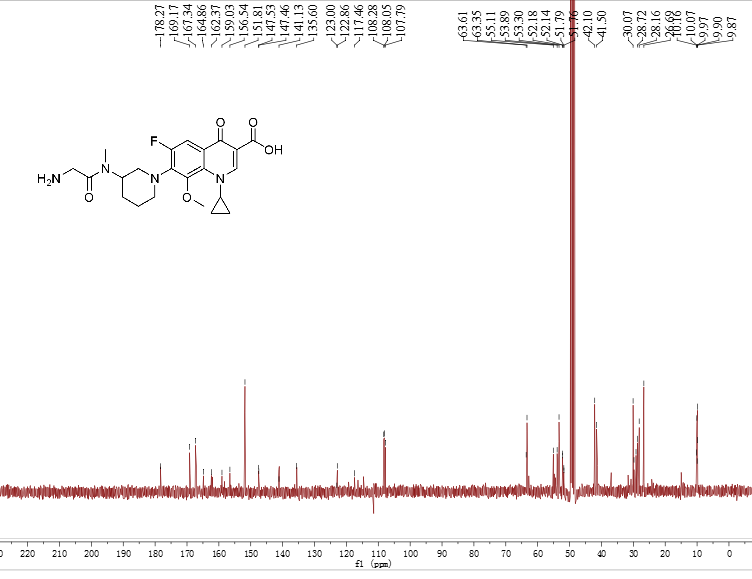


^13^C NMR Spectrum of Compound **20-e**

**
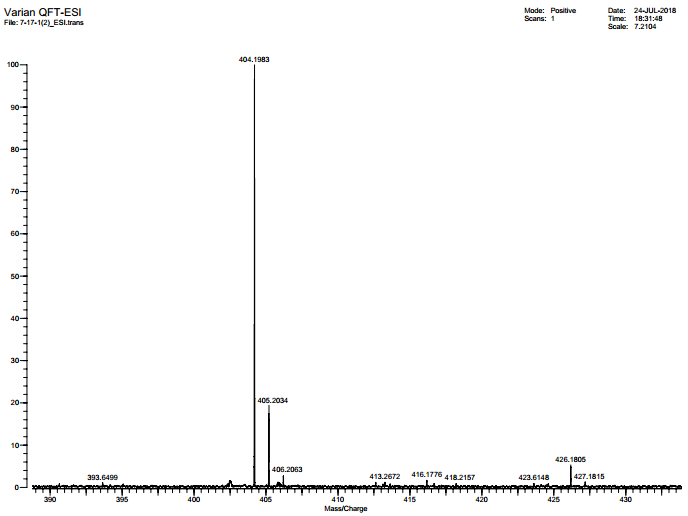
**

HRMS Spectrum of Compound **21-e**


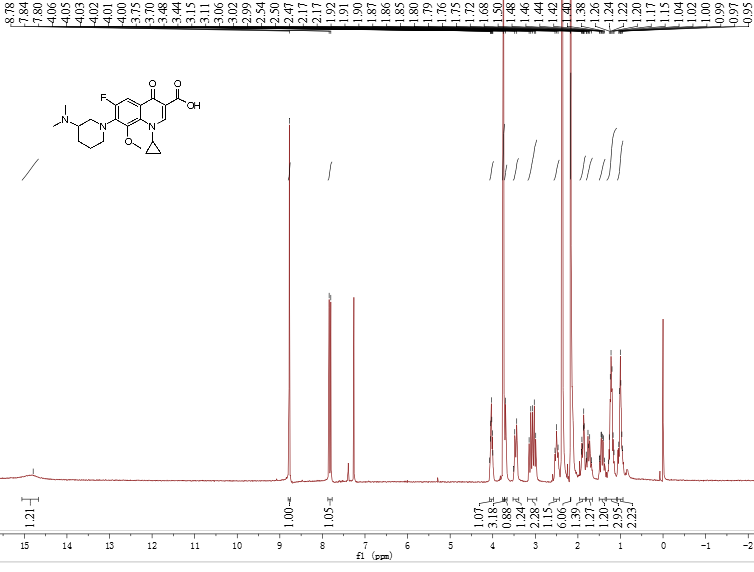


^1^H NMR Spectrum of Compound **21-e**


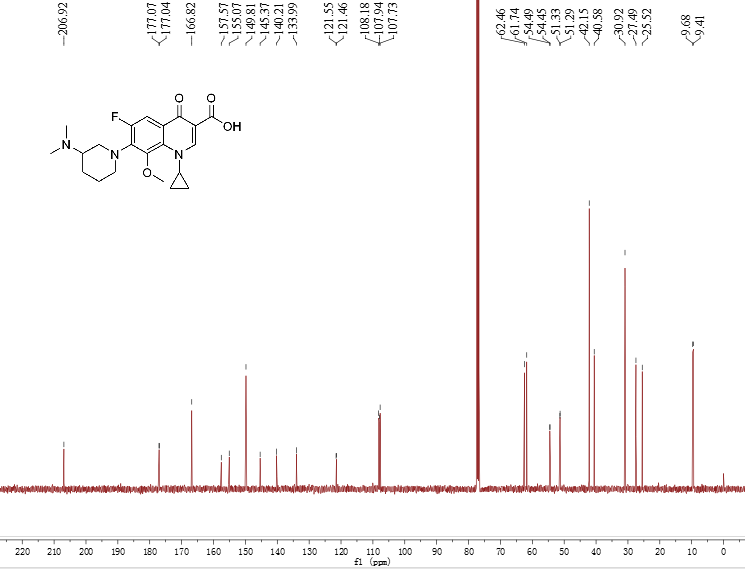


^13^C NMR Spectrum of Compound **21-e**


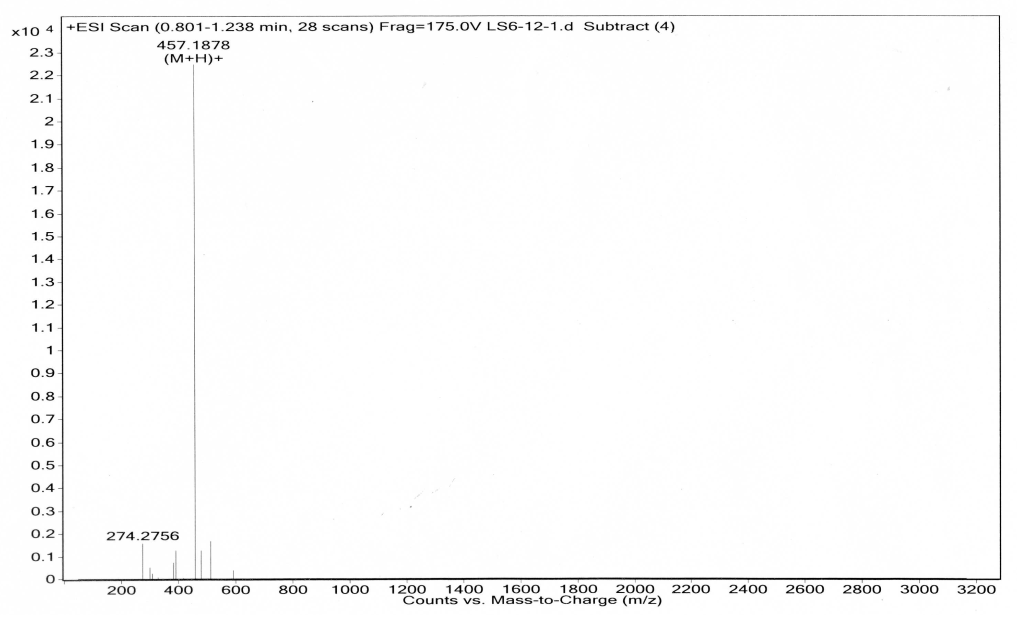


HRMS Spectrum of Compound **22-e**

^
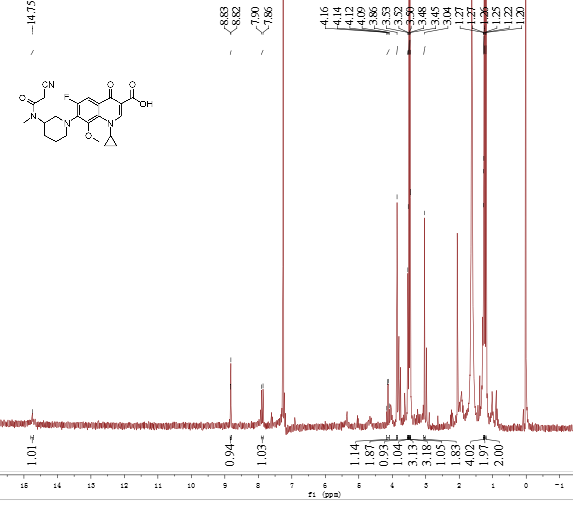
^

^1^H NMR Spectrum of Compound **22-e**
